# Mapping at-risk transportation infrastructure assets using statistical and machine learning methods

**DOI:** 10.1038/s41598-025-18440-w

**Published:** 2026-01-29

**Authors:** Rakesh Salunke, Sadik Khan

**Affiliations:** Department of Civil and Environmental Engineering, Jackson State University, Jackson, MS 39217 USA

**Keywords:** Susceptibility, Highway slopes, Machine-learning, GIS, Random forest, Infrastructure, Geotechnical asset management, Natural hazards, Civil engineering, Geomorphology

## Abstract

Geotechnical assets such as highway embankments and slopes (HWS) are critical to the integrity of transportation infrastructure. However, they are largely overlooked by Transportation Asset Management programs in the United States. The HWS are vulnerable to landslides induced by several factors including frequent occurrences of extreme rainfall events. Therefore, mapping vulnerable HWS and developing an inventory will significantly help with infrastructure asset management. To this end, this research adopted proven geographical information systems based on landslide susceptibility mapping methods typically applied to hillside slopes, and a method for mapping at-risk HWS assets was developed. Several supervised machine learning (ML) classification models were developed and evaluated to accurately map at-risk HWS in the study area of central Mississippi. Digital Elevation Models (DEMs) created from remote sensing data obtained from satellites, drone sensors, and terrestrial LiDAR were utilized to develop rasterized causative factors. The causative factors used included: Geotechnical and geomorphological attributes, such as slope, aspect, curvature, elevation, normalized difference vegetation index (NDVI), soil composition, and terrain from DEM; and hydrological factors, including precipitation, distance from the stream, groundwater depth, and topographic wetness index. Known locations of failed and not-failed HWS were selected and rasterized, and the pixels were extracted as ground truth data. The rasterized causative factors were utilized as independent features to train the classification ML models for predicting HWS failure susceptibility. Models were evaluated by developing confusion matrices and using probabilistic metrics such as area under curve (AUC) score, F-1score, and Accuracy scores. Random forest outperformed the other models (AUC, F1, and Accuracy scores of 1.0). Probability threshold tuning was performed on the random forest model, and susceptibility maps with different thresholds were evaluated. An optimal threshold of 0.75 was used to balance false negatives and false positives in the predicted results, ensuring more reliable identification of hazard-prone slopes. The trained RF model revealed that the elevation, distance from streams, the NDVI, and precipitation were the top four factors influencing HWS failures in this study. The method allows for easy identification of vulnerable HWS across vast geographic areas. This method helps in effective fund utilization by doing targeted interventions and preventative maintenance efforts. Transportation agencies can implement this methodology on HWS at any location to strategize geotechnical asset management efforts.

## Introduction

Geotechnical assets such as HWS, among other geo-structures, are integral to the multimodal transportation infrastructure. When they fail or experience problems, the overall infrastructure deteriorates. Annual repairs of failed slopes account for a significant percentage of the maintenance budget for many transportation agencies, including the Mississippi Department of Transportation (MDOT) and the US Army Corps of Engineers, and inaction will increase maintenance costs and lead to catastrophic failures without warning. Nevertheless, transportation asset management programs mostly focus on bridges and pavements^1–3^.

Natural hazards and increasingly frequent extreme rainfall events are exacerbating the deterioration of transportation infrastructure. Hurricanes like Harvey (2017), Florence (2018), Ida (2021), and Ian (2022) were more intense than previous ones and resulted in more devastating impacts, including causing landslides and impacting the transportation infrastructure. Mississippi’s transportation infrastructure network was devastatingly impacted by Hurricane Ida in 2021 and a record-breaking torrential rainfall in 2022^4^. In addition to the loss of lives, displaced communities, and property damage, the heavy downpours also caused significant geohazards, including landslides and washouts that impacted the transportation infrastructure. Landslide occurrences are expected to rise globally due to urbanization, deforestation, and precipitation changes^5^, impacting transportation infrastructure systems.

These transportation systems are crucial for economic development and regional connectivity. Disruptions caused by landslides, debris flows, and rockfalls not only hinder mobility but also lead to significant economic losses, endanger public safety, and complicate emergency response efforts.

The cascading impacts of these events on slope and embankment failures are profound, often crippling critical geo-infrastructure and disrupting essential systems such as transportation, energy, and water supply. Communities face severe setbacks, losing access to vital facilities like hospitals, shelters, and other critical infrastructure.

Landslide susceptibility mapping (LSM) using the Geographical Information System (GIS) is a proven methodology for understanding and forecasting the impacts of significant weather events on hillside cut slopes. Several studies have applied statistical models such as frequency ratio (FR) method^6–8^, weight of evidence (WOE) method^8,9^, and analytical hierarchical process (AHP)^10,11^ for LSM. WOE and AHP involve assigning scores to each influencing factor either randomly or based on experience. However, these methods rely on hypothesizing weights of influencing factors before modeling based on theoretical deduction and not empirical observations. To avoid such random weight assignments, several studies have implemented ML techniques^12,13^ such as support vector machines, decision trees, and random forests to assess landslide susceptibility^14–18^.

Lately, with readily available remote sensing data, researchers have increasingly applied GIS and ML methods to studying landslide susceptibility. Some of the studies in literature that have implemented LSM using GIS and ML worldwide, include Cameron Highlands area in Malaysia^11^, Isfahan province in Iran^19^, Mountain-side slopes of Karakoram highway in Pakistan^20^ Qingchuan county in China^21^, among others.^22^ highlight the critical role of ML in using remote sensing data for LSM and disaster risk management. Ma et al.^23^ also present an innovative application of Automated Machine Learning (AutoML) as a user-friendly, off-the-shelf tool that is very handy for LSM. However, many LSM studies targeted mountainous regions, focusing on landslides on natural or cut hillside slopes worldwide^11,19–21^. Notable exceptions include, Craig and Augusto Filho^24^ who implemented limit equilibrium methods to perform large-scale slope stability assessment along highway slopes in Sao Paulo, Brazil at a coarse scaled followed by a finer scale. Achour and Pourghasemi^25^ conducted a study that highlighted the potential of ML-based LSM for identifying high-risk zones along critical highway infrastructure in mountainous regions of Algeria. Their study found that Random Forest outperformed (AUC = 0.97) the other models in LSM.

However, relatively little attention has been given to slope instabilities affecting slopes in low-relief or planar terrains. Particularly, there remains a notable gap in the literature concerning embankment slope failures affecting transportation infrastructure, such as highways, railways, and intermodal transportation networks. This gap is especially pronounced in regions like Mississippi, where expansive clays and alternating patterns of intense rainfall and drought, often intensified by hurricanes, contribute to shrink-swell cycles that undermine the stability of highway and railroad embankments. These geotechnical instabilities threaten the long-term performance, safety, and serviceability of infrastructure assets, highlighting the urgent need for targeted susceptibility modeling. Despite their importance, earthen embankment slopes remain underrepresented in mainstream LSM research.

Although a growing number of researchers strongly recommend implementing remote sensing data to monitor and manage geotechnical and transportation infrastructure assets such as highway embankments and slopes^26–30^. However, there have been few studies implementing LSM focused on highway slopes using GIS and remote sensing techniques. In this age of information and AI, researchers must harness remote sensing techniques with AI to monitor geotechnical assets such as embankment slopes and, by extension, transportation infrastructure.

Therefore, in this current study, we address the research gap and develop a HWS failure susceptibility map along the slopes of Mississippi’s highway corridor using GIS and an ML-based framework. This research seeks to bridge existing knowledge gaps by conducting a detailed evaluation of highway embankments and their vulnerability to slope failures in Mississippi. The process involves creating an inventory of both failed and intact highway slopes, followed by the application of ML techniques for susceptibility mapping. Various supervised ML models, including random forest, naïve Bayes classifiers, support vector classifier, and logistic regression, are assessed to determine their effectiveness in predicting highway embankment and slope failure susceptibility. Although there is no consensus on which ML model should be used to perform LSM^31^, the models used in this study have been extensively used by other LSM studies. The models were evaluated by comparing the Accuracy, F-1score, and AUC scores, which are popular performance metrics for LSM classification models^32^.

Various data sources were utilized to develop causative factors, including DEMs created from remote sensing methods such as satellites and aerial LiDAR. The LSM focused on highway slopes is aimed at contributing towards enhancing infrastructure resilience by enabling proactive monitoring, early warning, and risk-informed asset management through the integration of geospatial analysis, remote sensing, and predictive modeling.

The causative factors considered in this study included geotechnical and geomorphological attributes, such as the slope’s inclination angle, aspect, curvature, the normalized difference vegetation index (NDVI), and the soil type. Hydrological factors, including precipitation, distance from streams, the topographic wetness index (TWI), and groundwater depth, were also incorporated. The selected causative factors have been consistently used in landslide and geohazard susceptibility modeling studies by several studies^10,11,33–36^. Further detailed discussion on the selection of causative factors is provided in the Methods section.

The causative factors were utilized as independent features for training the ML classification models to predict vulnerable highway slopes and embankments. The results of this study can significantly contribute to the work of transportation agencies and authorities by providing valuable insights for targeting preventative maintenance efforts on geotechnical assets and mitigating catastrophic failures caused by significant rainfall and weather events on road networks and highway slopes.

Although developing failure susceptibility models using GIS combined with ML models is a proven technique, it has rarely been applied to map unstable highway embankment slopes. The GIS-based susceptibility modeling technique proposed in this study will be instrumental in developing an inventory of at-risk highway slope assets. The study’s framework has broader applications, extending to railroad embankments and other critical geotechnical infrastructure, such as levees and earthen dams, which form essential components of intermodal transportation systems.

## Methods

The study was accomplished in the following stages: (i) development of a failed and non-failed embankment slopes inventory database; (ii) determining the causative factors; (iii) building and comparing ML classification models for predicting failure, including random forest, logistic regression, Naïve Bayes and SVC; (iv) generation of a failure susceptibility map of unstable slopes and embankments using the best performing ML model.

**Fig. 1:**
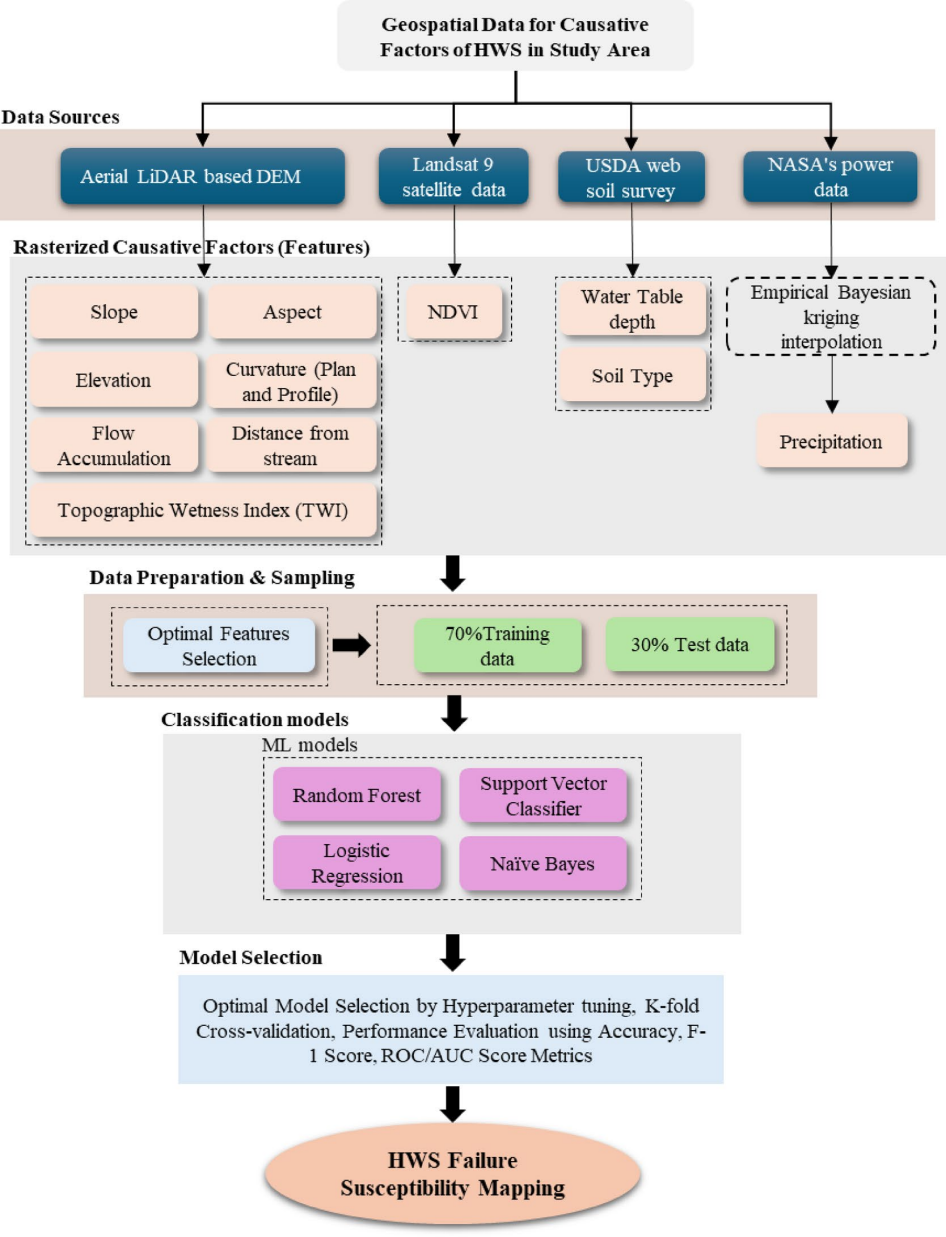
Methodology schematic for HWS Failure Susceptibility Mapping. *Note*: Landsat 9 satellite imagery^37^ obtained from U.S. Geological Survey Earth Explorer (earthexplorer.usgs.gov/) were used to create NDVI raster by the first author (R.S.). All rasterized causative factors and the HWS susceptibility maps were created in ArcGIS Pro software by Esri, version 3.0 (https://www.esri.com/arcgis) by the first author (R.S.)

The procedure was carried out by first preparing causative factor maps in raster format, which is a popular and computationally efficient way of conducting LSM^23^. The methodology is described in the schematic presented in Fig. 1.

The following procedure was followed to accomplish the susceptibility mapping.

- Step 1: An inventory of failed and non-failed embankments within the study area was developed. The locations of the embankments were vectorized in Google Earth and saved as a kmz file, then inserted in ArcGIS, converted to failed and non-failed polygons, and then rasterized.
- Step 2: Causative factors most likely influenced the failure were developed as rasters. Topographic, geomorphological, and hydrological factors were developed using DEMs obtained from various sources.
- Step 3: The data was analyzed, the models were developed, and the classification test results from various ML models were compared.
- Step 4. The better-performing model (Random Forest) was used to generate failure susceptibility maps of the highway embankment slopes along the Major Mississippi network corridor.

The software used for susceptibility mapping exercises included ArcGIS Pro software by Esri, version 3.0 (www.esri.com/arcgis), open source Python libraries including the Pandas library version 1.5.3

(pandas.pydata.org), Scikit-learn version 1.2.2 (sklearn, scikit-learn.org/), and Geospatial Data Abstraction Library “GDAL” version 3.2.4 (gdal.org). All analyses were carried out in Python in JupyterLab 3.6.3 (jupyter.org), an interactive web-based development environment in the Anaconda Navigator distribution platform 2.5.0 (www.anaconda.com). The Aerial LiDAR based DEMs were obtained from Mississippi Automated Resource Information System (MARIS, maris.mississippi.edu/) made available to the public by the State of Mississippi. The DEMs were developed using data collected by aerial LiDAR survey missions conducted during 2005–2017. The DEM was in the horizontal projection system: NAD 1983 (2011), UTM 15N and 16N, Vertical Datum: NAVD88 GEOID12b, with a resolution or pixel spacing of approximately 0.7 m (or 3 ft.). Such high-resolution DEMs are particularly valuable because they provide finer spatial detail and can increase the accuracy of susceptibility maps^38^ compared to lower-resolution satellite data, such as the 30 m or 98 ft resolution from Landsat 9, for example.

### Study area and dataset

#### Highway embankment and slope inventory

**Fig. 2:**
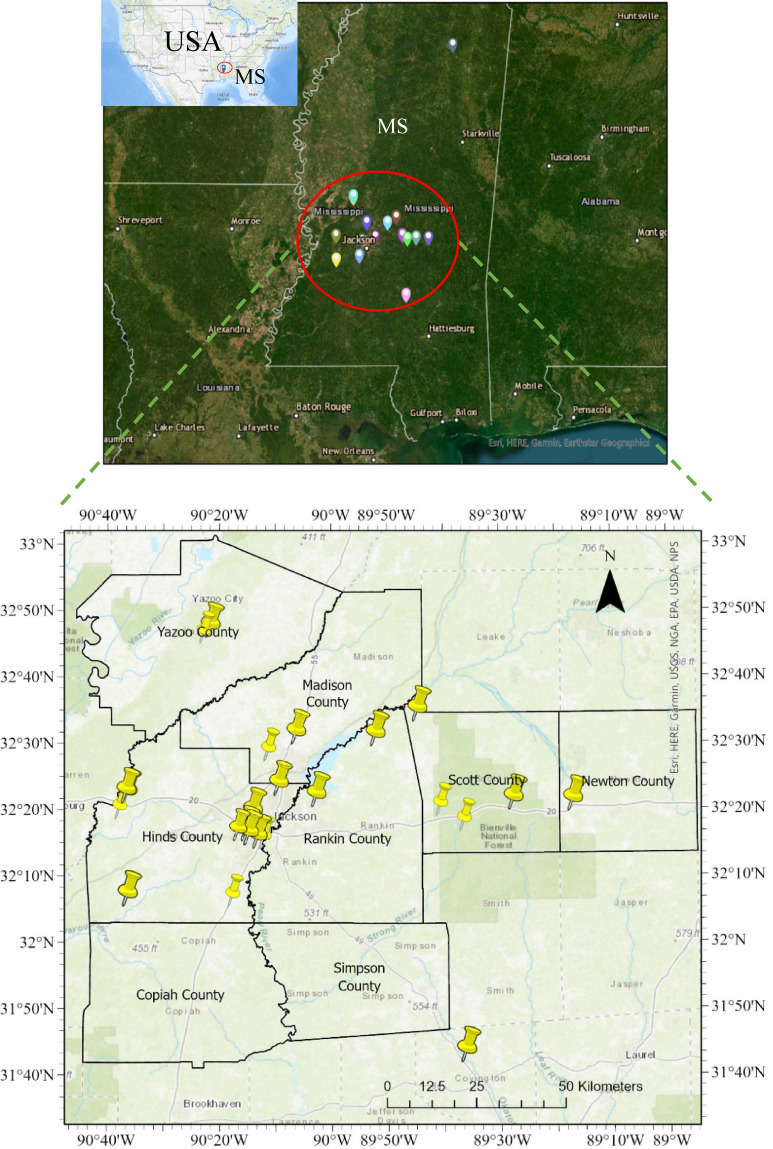
Study area location and HWS inventory map generated in ArcGIS Pro software (Esri, version 3.0, www.esri.com/arcgis) by the First Author (R.S.)

**Fig. 3:**
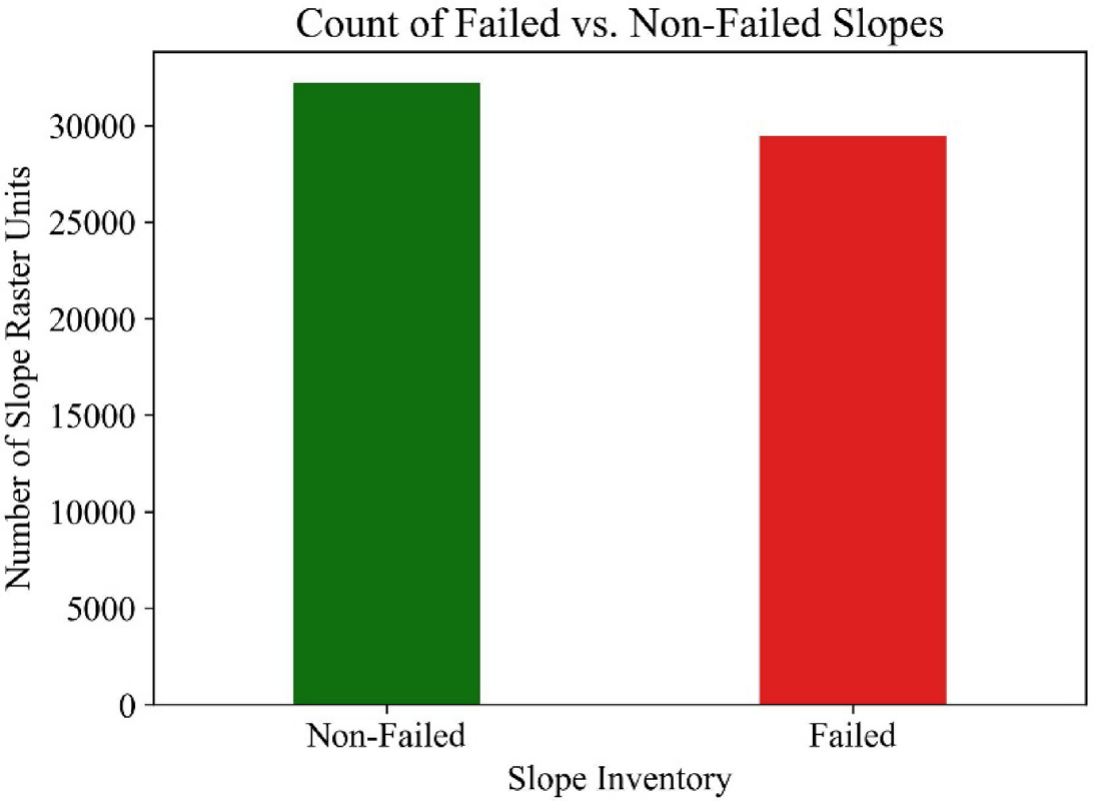
Slopes inventory distribution.

The failed slopes investigated in this study were from eight counties in Mississippi. There were approximately 64,000 pixels of failed slope areas at a resolution of 0.9 m. × 0.9 m., representing an area of 0.83 sq. m. per pixel. Approximately double the number of pixels representing non-failed areas was randomly selected within the study area to develop the failure susceptibility prediction models. The failed and non-failed HWS locations are presented in Fig. 2. The non-failed HWS identified by polygons is not visible on the map in Fig. 2 due to the large scale of the map. The count of Failed vs. Non-Failed Slopes is presented in Fig. 3.

### Causative factors dataset preparation

The most important step in model development for slope failure susceptibility mapping is the preparation of the causative factors dataset used as features for model training and validation. In general, the accuracy of models is influenced by data sampling strategies. Failed embankment slopes were sampled as grid cells, a proven method used for natural hazard susceptibility modeling, including landslides^39^. The failed embankment slope polygons were imported in kmz format and converted to rasters in ArcGIS Pro. Then, the raster was sampled to a pixel grid size of 3’ × 3.' Out of the 26 failed slope sites in the study area of the eight counties (Fig. 2), comprising 29,469 grid cells, were used for susceptibility modeling. Non-failed embankment slope locations with a proportion of 1:2 (or twice the number of failed slopes) were randomly selected within the study area. The failed slopes were assigned a score of 1, while the non-failed slope pixels were assigned a score of 0. Both failed and non-failed slope rasters were used to sample the causative factors. By doing so, a dataset consisting of the 14 columns of features and a target column of failure classification with 0 or 1 value was developed. After cleaning and optimizing the data, the total dataset shape consisted of 32,217 rows and 15 columns. The causative factors and dataset preparation were carried out in ArcGIS Pro and the JupyterLab interface in Anaconda Navigator, using Python and the GDAL library.

The selection of causative factors was based on an extensive literature review as described in Table 1. The most common and relevant causative factors were selected, namely: elevation, slope, etc.^38^ found that increasing the number of causative factors from 12 to 15 increased the prediction accuracy slightly. Therefore, we started by using 15 causative factors, namely: elevation, slope, aspect, curvature, plan curvature, profile curvature, NDVI, TWI, Distance from Streams, Flow Accumulation, Water Table Depth, and Soil Type. The commonly used causative factors in other studies obtained from literature review are described in Table 1. Each causative factor used in this study is discussed in the section ahead.

**Table 1:** Causative factors for LSM in previous studies.

Causative factors	Studies
Elevation/altitude	^20,23,32,38,40–44^
Hill slope/Slope	^5,11,20,32,38,40–43,45,46^
Slope aspect/Aspect	^5,11,20,32,38,40–43,45,46^
Lithology/Soil/Rock Group	^11,20,23,32,40–43^
Distance to faults	^11,20,32,41,43^
Distance to streams/rivers/drainage	^11,20,23,32,38,40–43^
Distance to roads	^11,23,32,41–43^
NDVI	^11,20,40,41,43,47^
Land cover/Land use	^11,32,42^
Bedrock lithology/types	^42^
Curvatures (plan, profile, tangential)	^23,38,40,45^
TWI, SPI	^23,32,38^
Precipitation/MAP	^11,20,32,43^
Vegetation type/extent	^44,48,49^
Flow length/accumulation, Solar radiation	^38^
Epicenter/Seismogenic fault, PGA	^23^

#### Elevation

**Fig. 4:**
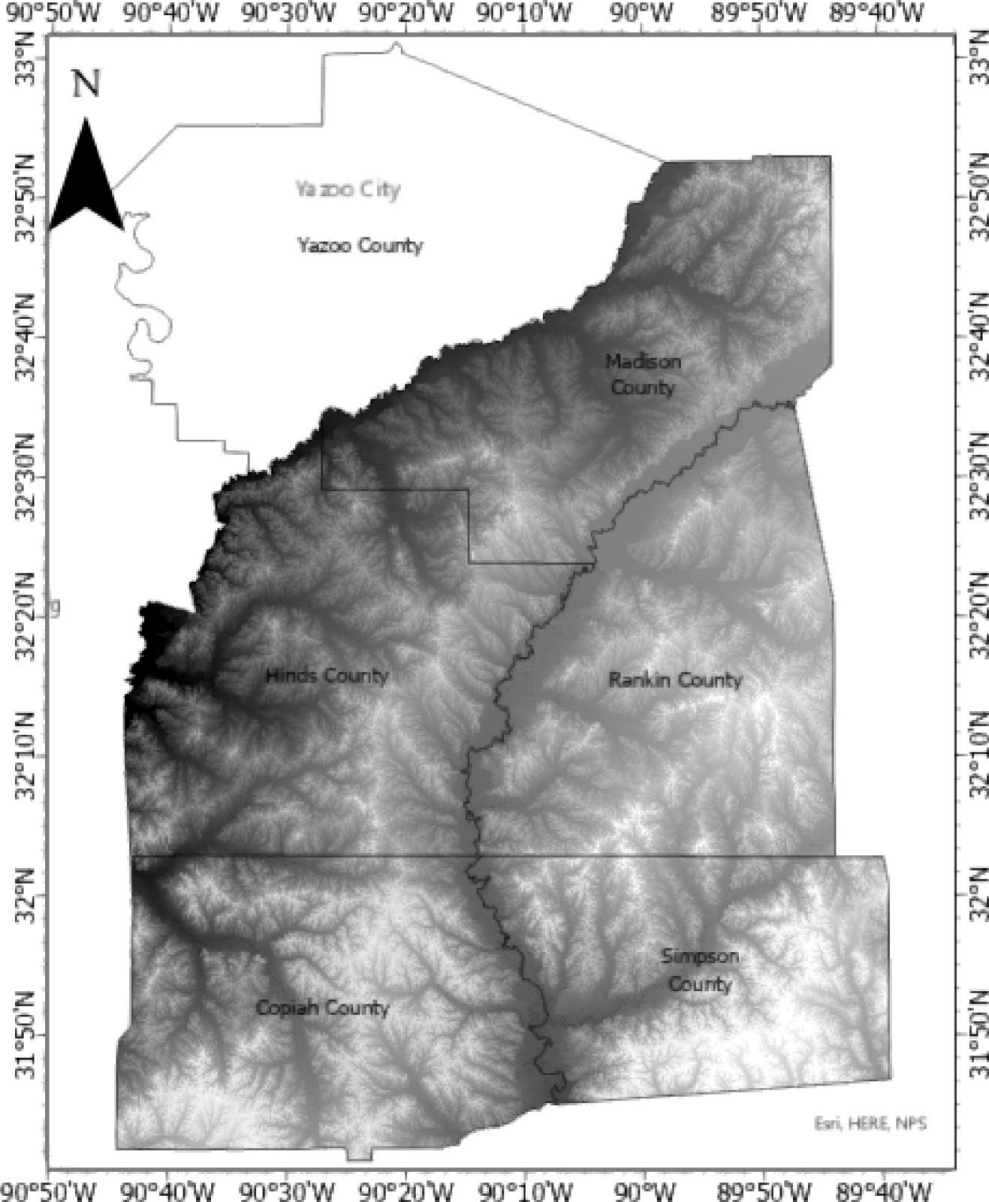
DEM for Study Area (resolution = 0.7 m or 2.3ft.). Data source: Aerial LiDAR based DEM publicly available from MARIS (maris.mississippi.edu).

Elevation is a crucial contributing factor frequently employed in evaluating landslide susceptibility^50^. The study area’s DEM exhibits a spatial resolution of 0.7 m. Figure 4 illustrates the MARIS-derived DEM for the study area.

**Fig. 5:**
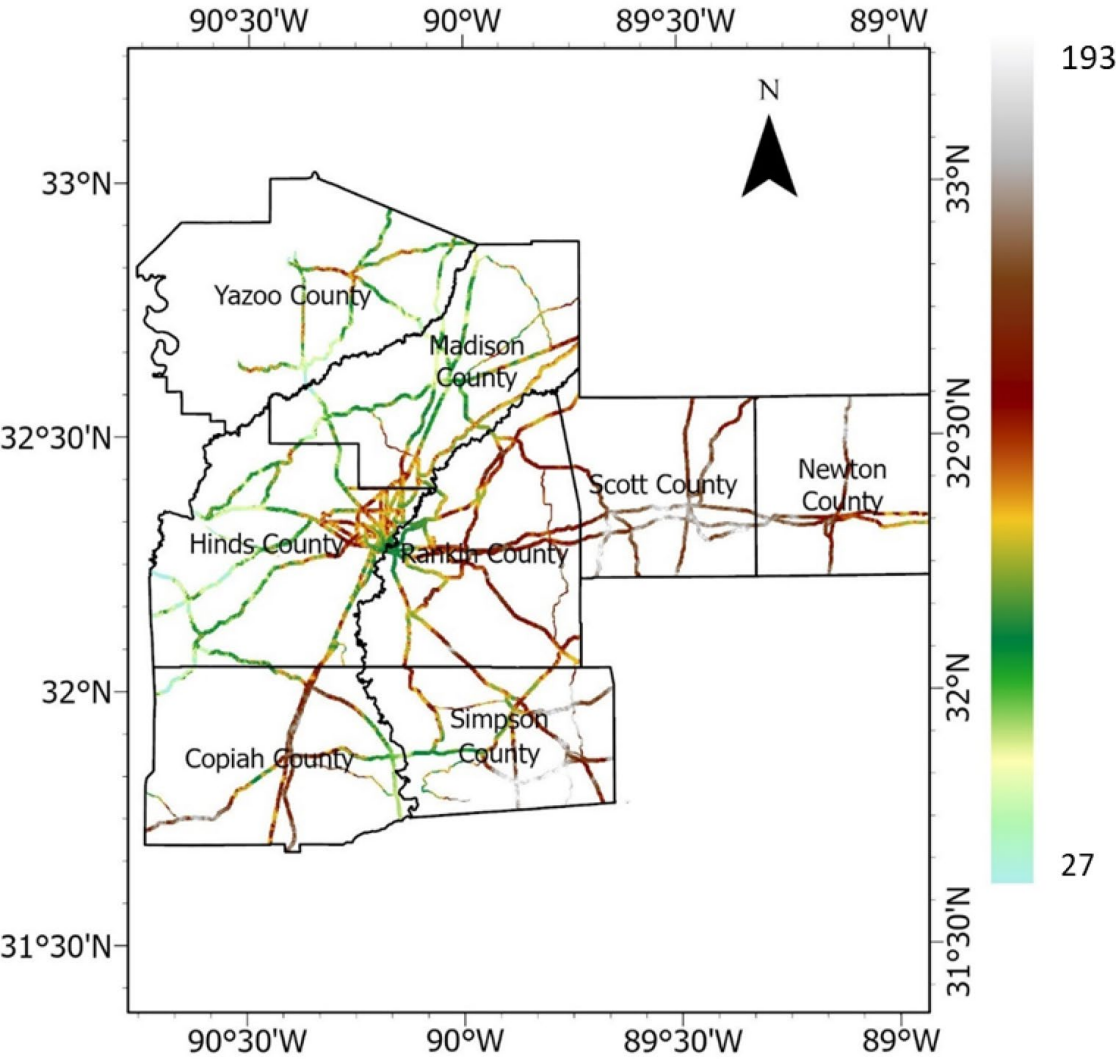
Elevation raster (meter).

National highway corridors, state highways, and roads were selected and vectorized in Google Earth, and a buffer polygon of up to 427 m. was generated on both sides of the roads. Subsequently, using the buffer zone as polygons, the DEM was clipped to generate the raster representation of the highway embankment, as depicted in Fig. 5. The elevation varied from 27 to 193 m. This DEM of the highway embankment was used to create other rasterized causative factors, including slope, aspect, TWI, curvature, plan and profile curvatures, flow accumulation, and distance from a stream.

#### Slope

**Fig. 6:**
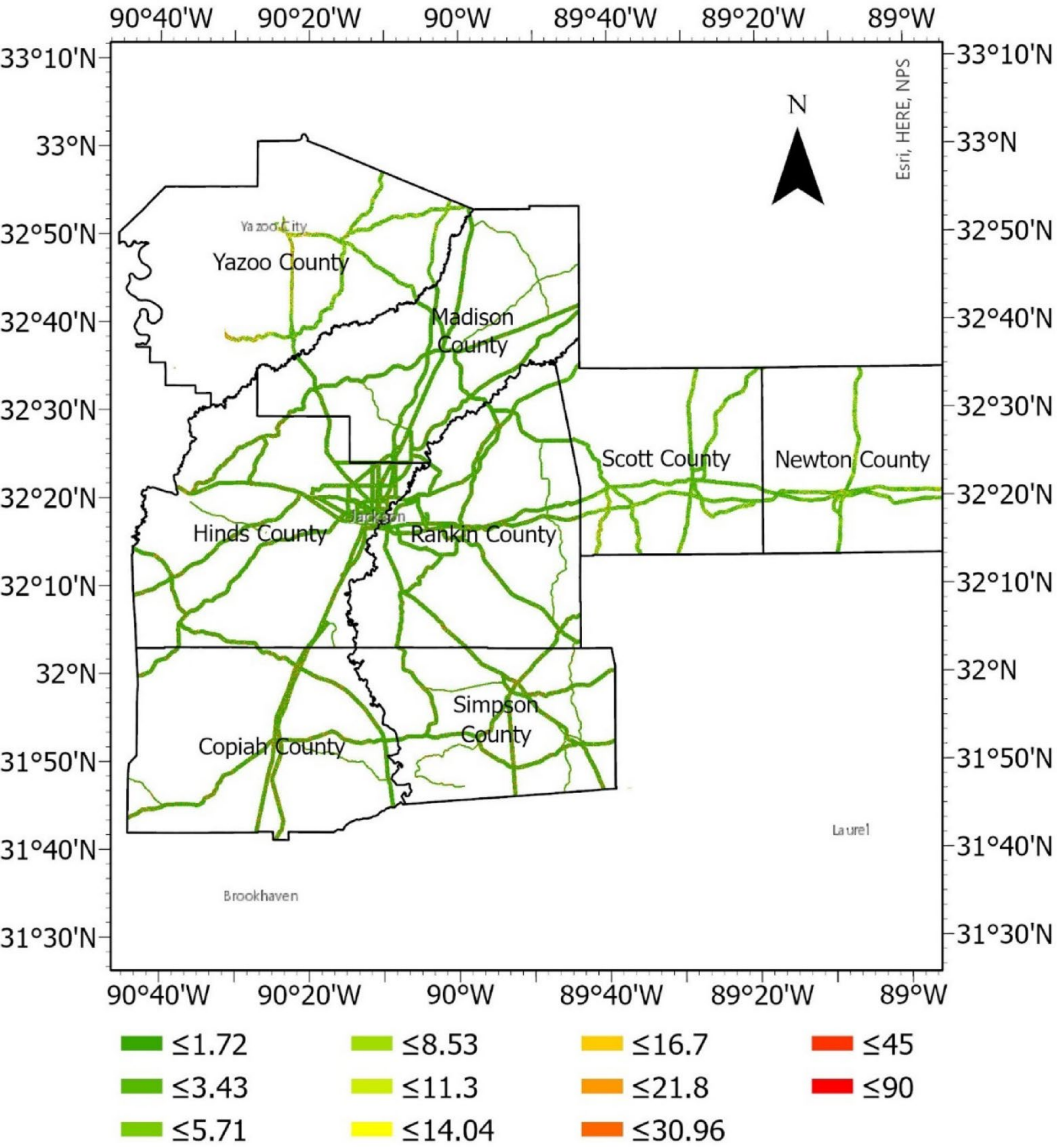
Slope raster (degrees).

Slope (or grade) is the steepness or inclination of a land surface. It plays a pivotal role in influencing the stability and susceptibility of terrain and is a critical parameter in understanding the behavior of landscapes and geological formations. The slope angle is typically measured in degrees or radians and is a crucial factor in various natural and engineered environments. The slope raster in degrees is presented in Fig. 6.

#### Aspect

**Fig. 7:**
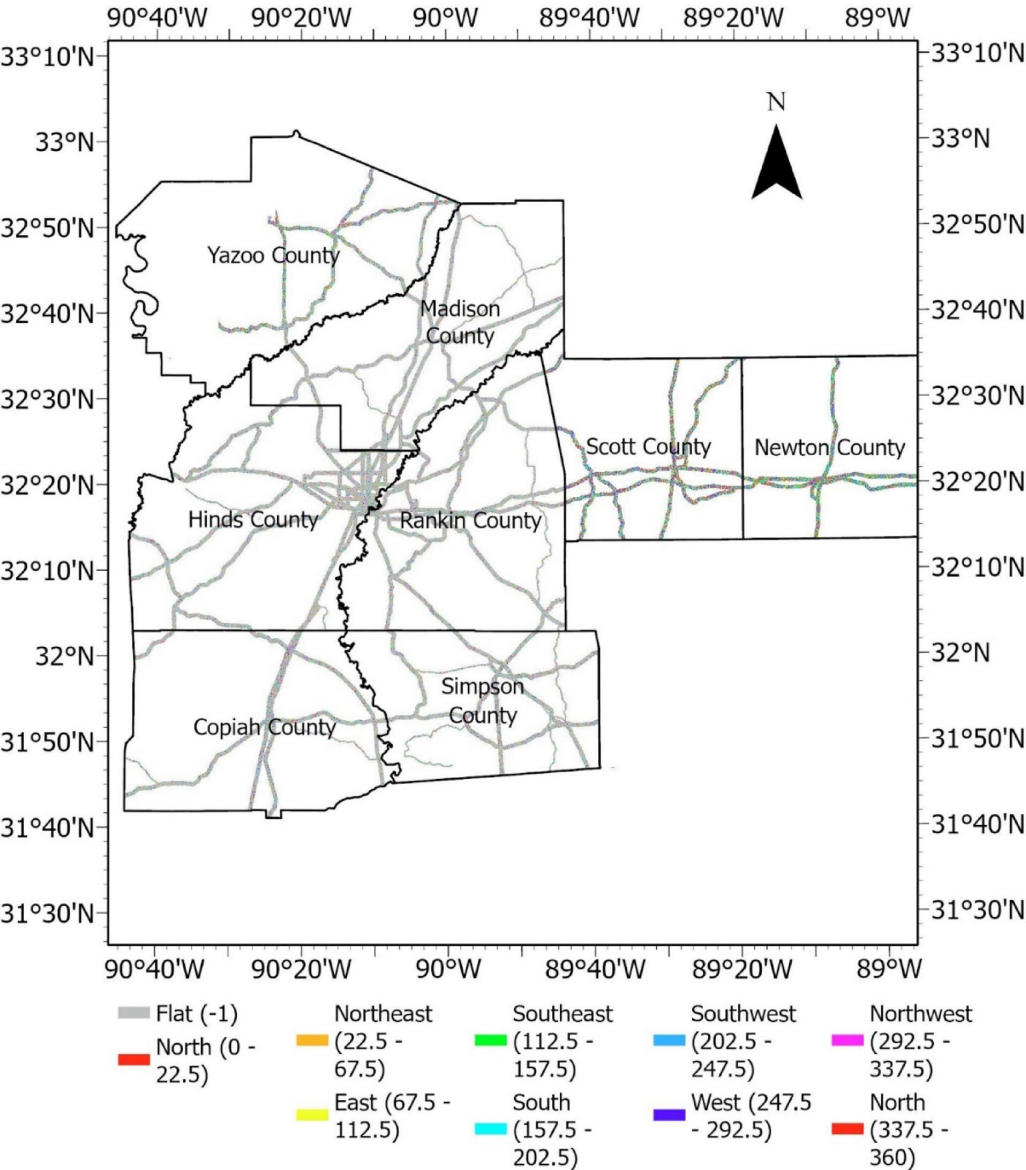
Aspect raster.

Aspect indicates the slope facing direction. It measures the orientation of the steepest downhill path on a slope and is often expressed in degrees, indicating the direction a slope faces with respect to due north. It is a fundamental component in terrain analysis and influences landslide susceptibility by affecting exposure to sunlight, temperature variations, evapotranspiration, precipitation impact, and erosion^32^. Aspects are usually categorized into cardinal directions (north, south, east, or west) or are specified in degrees, providing valuable information for various applications, including agriculture, forestry, hydrology, and geotechnical engineering. The Aspect raster is presented in Fig. 7.

#### Curvature

**Fig. 8:**
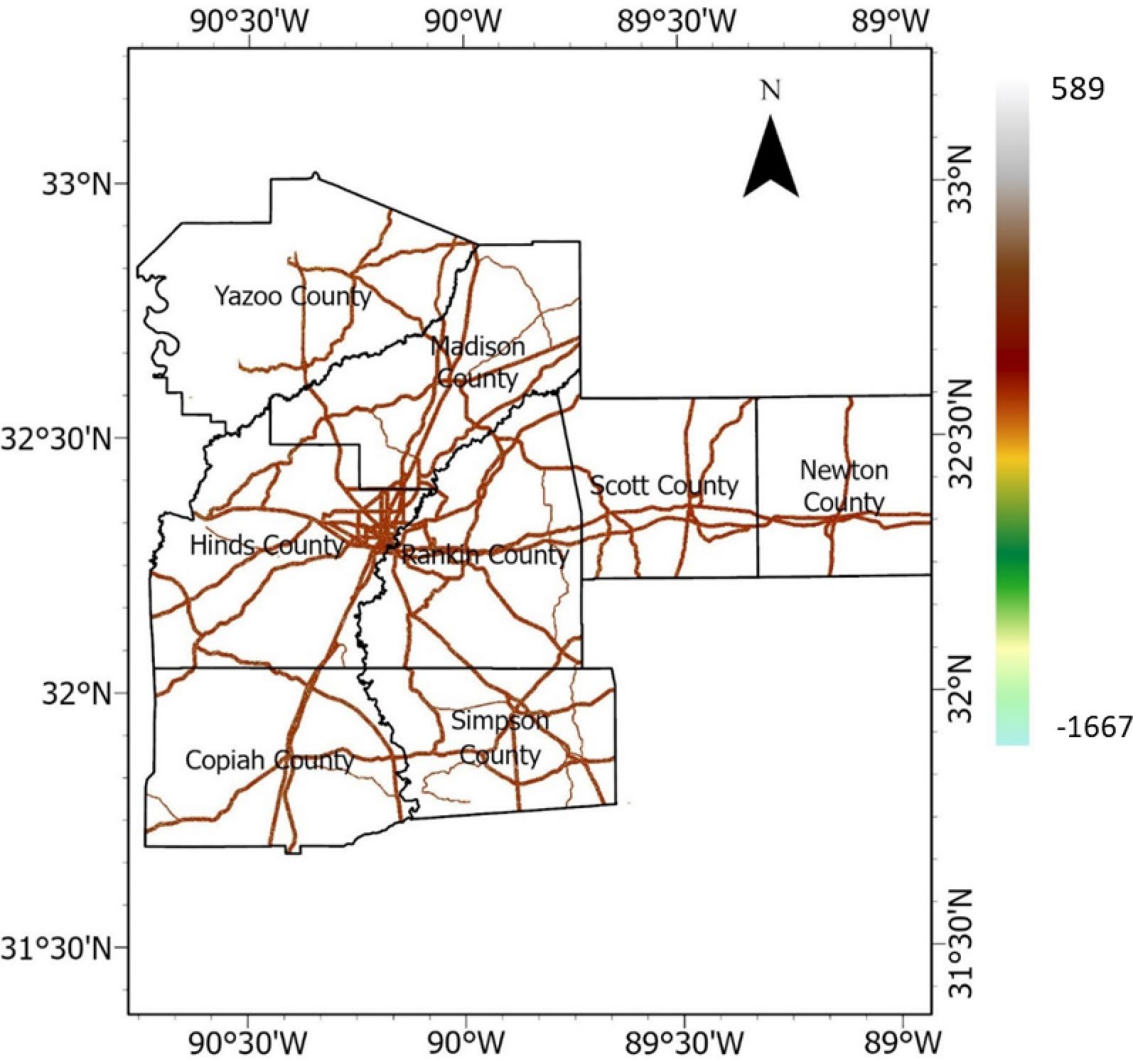
Curvature raster (meter).

Curvature refers to the rate of change of a slope along a surface. It is a key factor in understanding geomorphology, as it indicates whether a surface is concave or convex and provides insights into the overall shape of the terrain. There are two main types of curvature: plan curvature and profile curvature. The curvature raster for the study area is presented in Fig. 8.

**Fig. 9:**
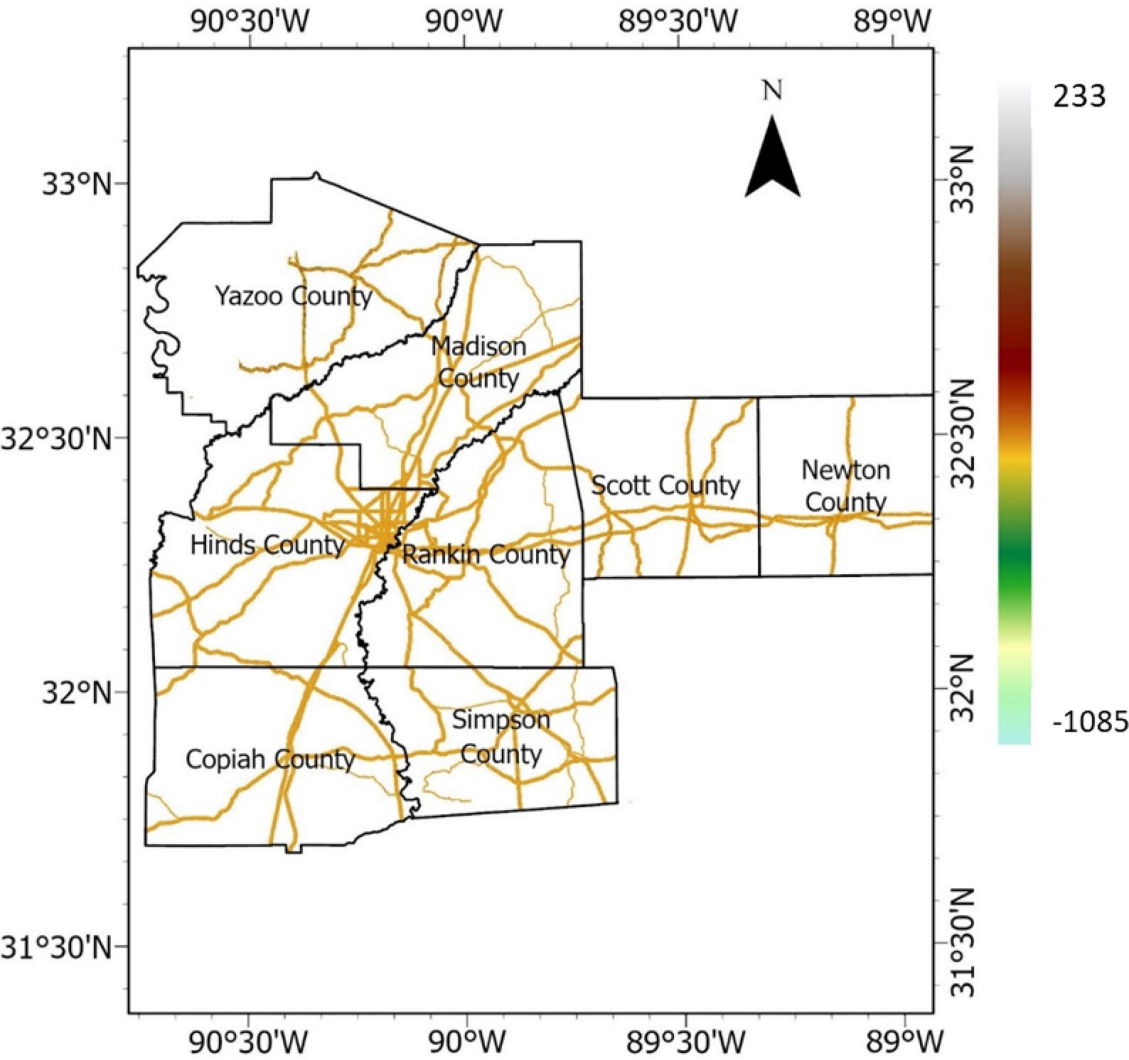
Plan curvature raster (meter).

**Fig. 10:**
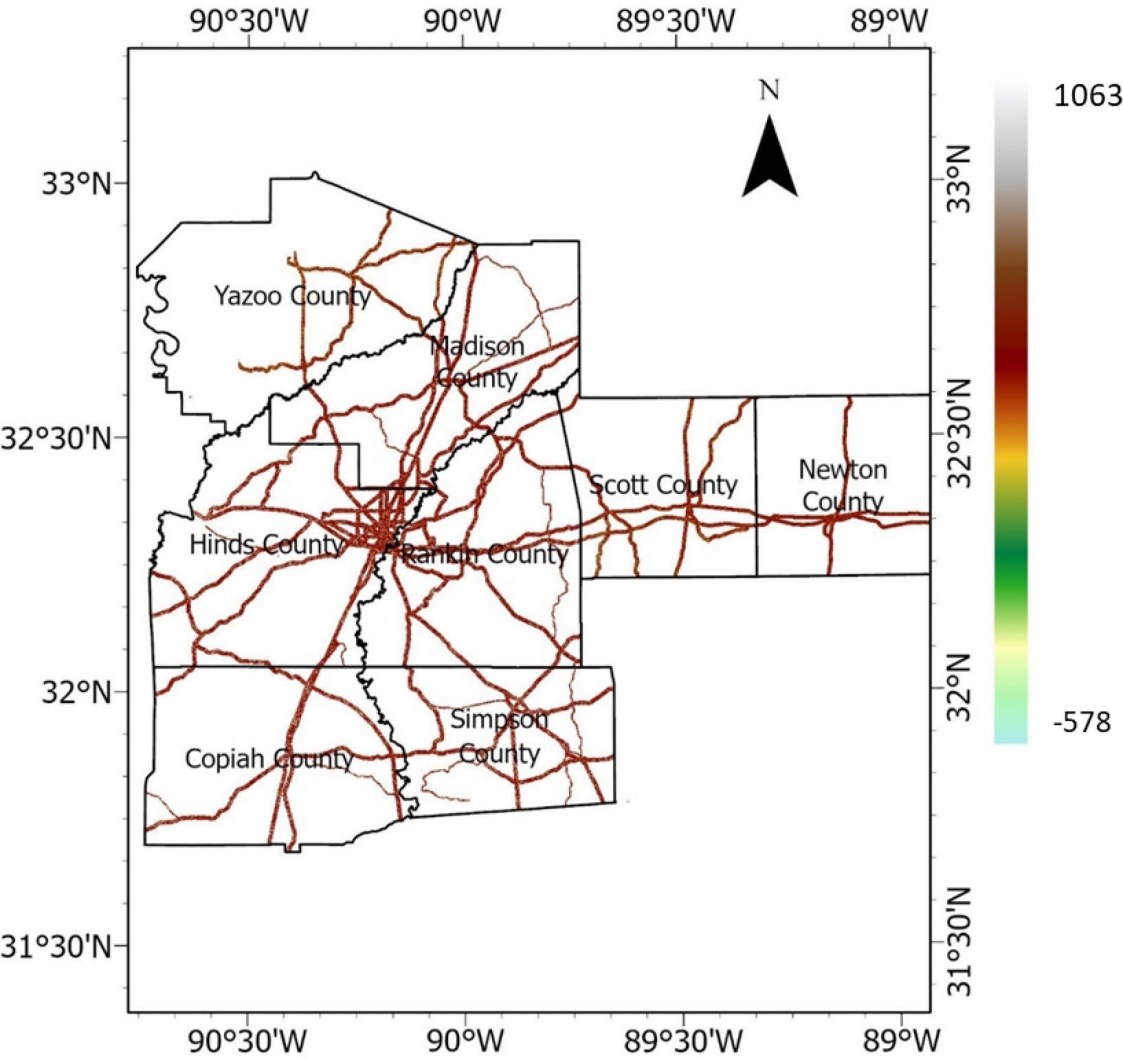
Profile curvature raster (meter).

- *Plan Curvature:* A plan curvature, also known as a contour curvature, represents the curvature perpendicular to the slope’s direction and indicates how a surface curves when it moves parallel to the contours. Positive plan curvature indicates convexity, while negative plan curvature indicates concavity along the contours. The plan curvature raster for the study area is presented in Fig. 9.
- *Profile Curvature:* A profile curvature represents the curvature parallel to the slope or along the steepest slope direction. A positive profile curvature indicates upward curvature (concave-up), while a negative profile curvature indicates downward curvature (concave-down). The profile curvature raster for the study area is presented in Fig. 10.

#### Normalized difference vegetation index (NDVI)

NDVI is a widely used vegetation index in remote sensing and is particularly valuable for assessing vegetation health and density. Healthy vegetation means the roots are strong and hold the near-surface soil together, improving the soil’s shear strength. NDVI can be an influencing factor for embankment and slope failures. The formula for NDVI is presented in Eq. (1).

1 $$NDVI = \frac{NIR - Red}{{NIR - Red}}$$

where, NIR = Near Infrared reflectance, and Red = Red reflectance value

**Fig. 11:**
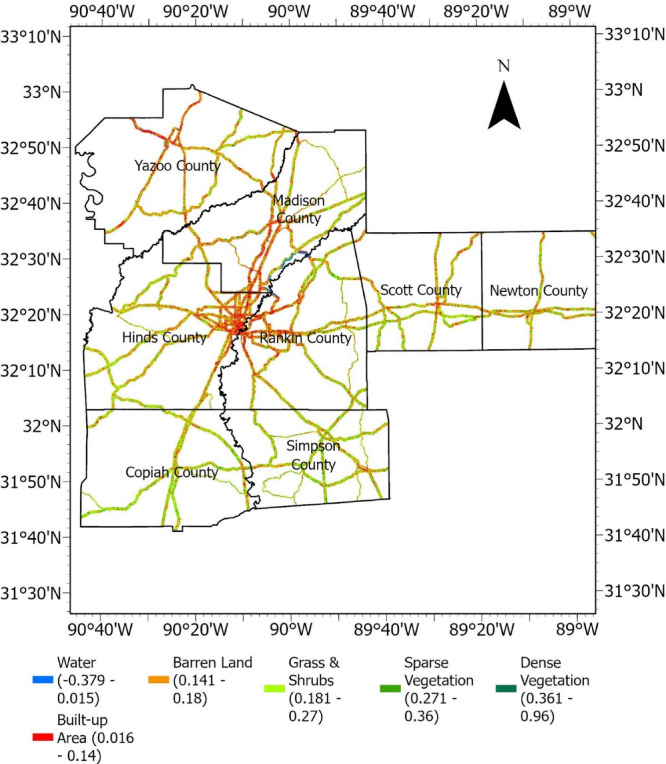
NDVI raster.

Landsat 9 satellite imagery^37^ obtained from U.S. Geological Survey Earth Explorer (earthexplorer.usgs.gov/) were used to create NDVI raster. The multiple spectral bands of Landsat 9, including the red and near-infrared bands, were extracted in ArcGIS Pro, and NDVI was calculated using the above equation. NDVI values typically range from − 1 to + 1; water bodies are close to -1; barren areas, rocks, or built-up environments are close to 0; and values of 0 to 1 are assigned to areas based on increasingly dense and healthy vegetation. The NDVI raster developed for the study area is presented in Fig. 11.

#### Topographic wetness index (TWI)

**Fig. 12:**
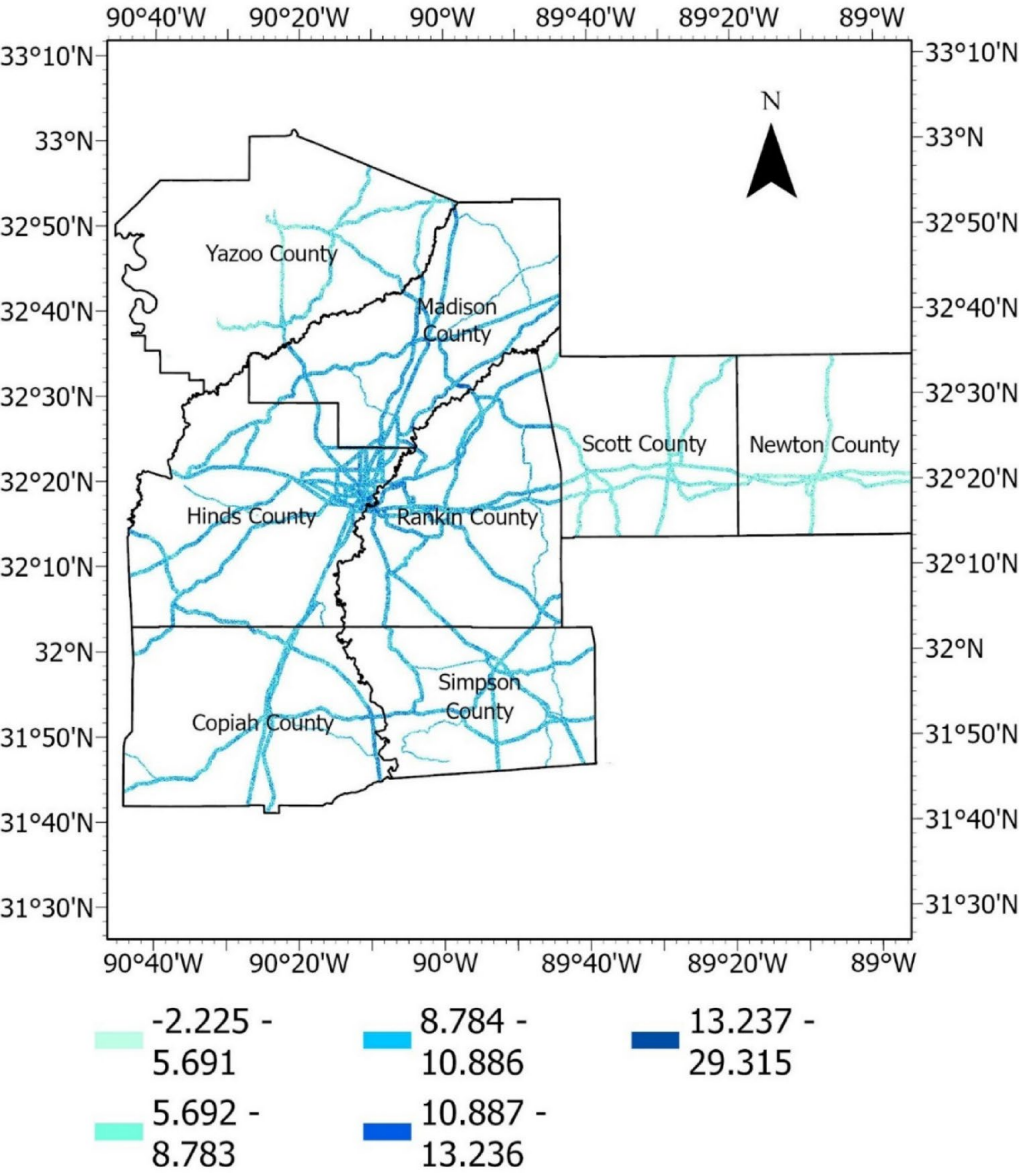
TWI raster.

The topographic wetness index is a dimensionless index calculated from DEMs. It considers each cell’s slope and contributing area within a study area. Higher TWI values generally indicate areas with increased potential for water accumulation, while lower values suggest better drainage. Areas with higher TWI might be more prone to saturation and increased pore water pressure, potentially contributing to slope instability or embankment failure. The TWI is often calculated using the formula in Eq. (2).

2 $${\mathrm{TWI}} = {\mathrm{In}}\left( {\frac{a}{{\tan (\beta )}}} \right)$$

where, a is the specific contributing area (upslope area draining to a point per unit contour length), β is the slope in radians. The TWI raster is presented in Fig. 12.

#### Flow accumulation

**Fig. 13:**
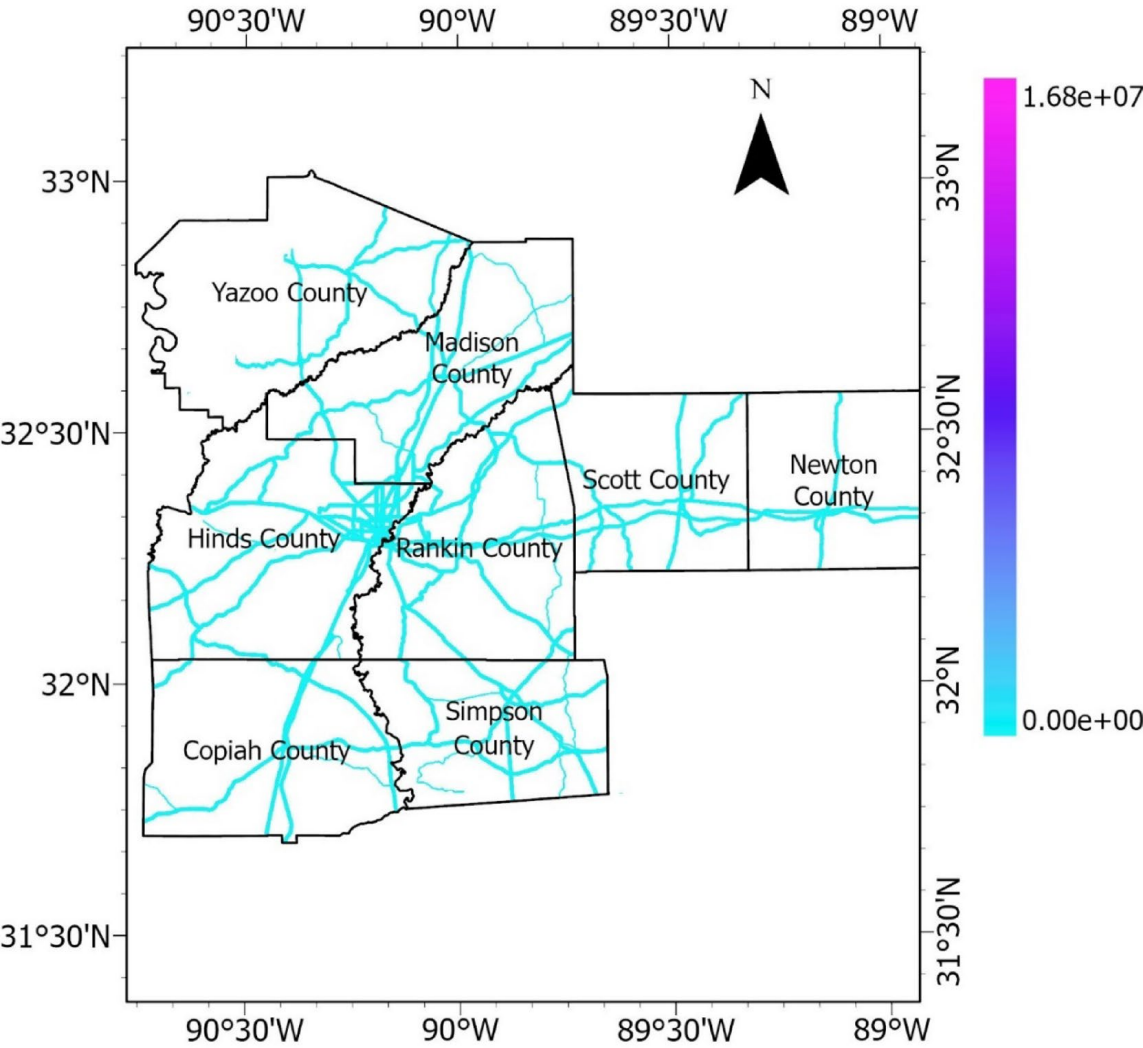
Flow accumulation raster.

Flow accumulation refers to the number of cells contributing flow to a specific cell in a raster grid. Each cell displays a value representing the total number of cells draining into the cell. In the context of hydrology, it represents the accumulated upstream area draining into each cell, indicating the potential flow or runoff. The flow accumulation raster is presented in Fig. 13.

#### Precipitation

**Fig. 14:**
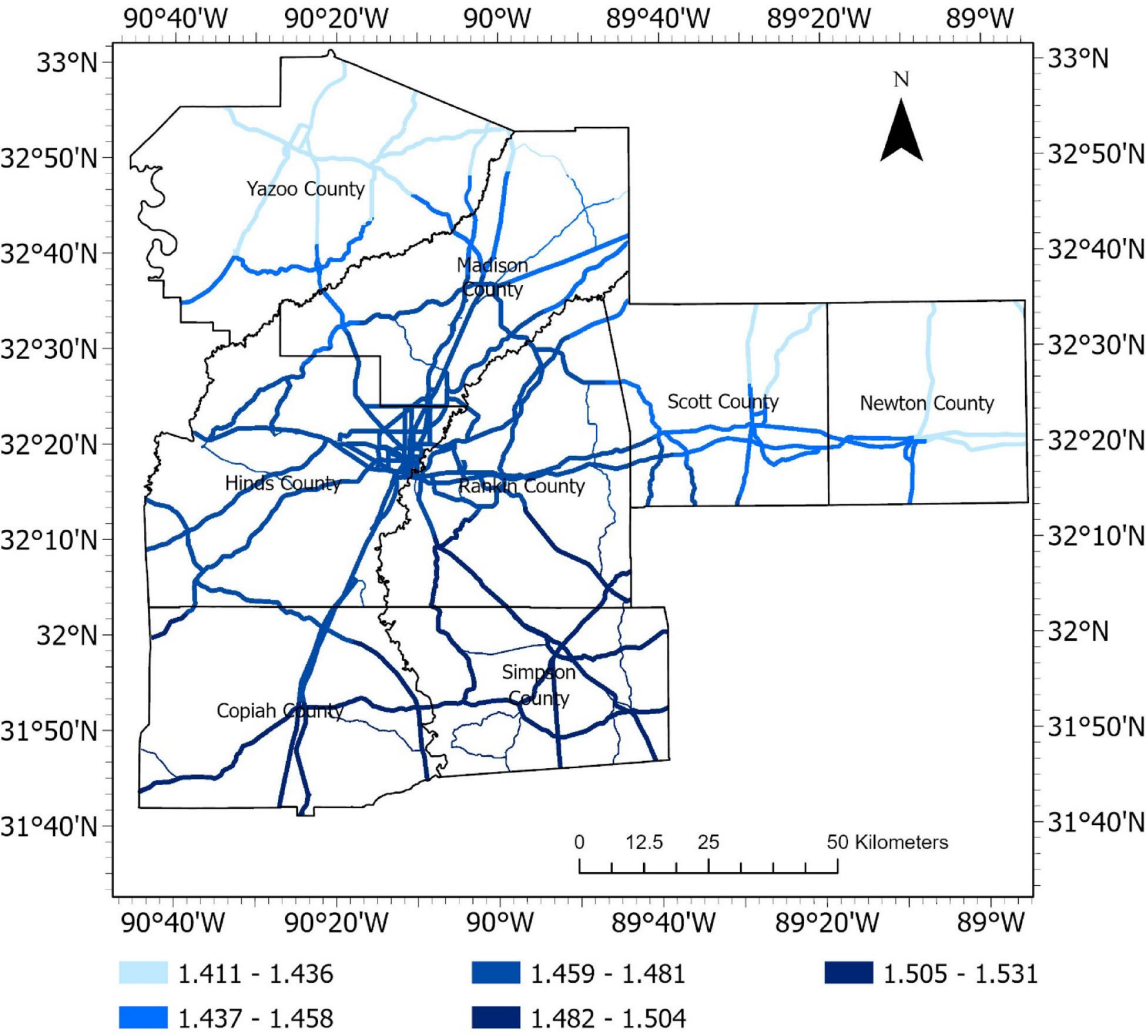
Precipitation raster (meter).

Precipitation is a crucial triggering factor of landslides and HWS failures. Rainfall data between 2011 and 2020 were downloaded from NASA’s power data access website for the eight Mississippi counties in the study area, and the averages were calculated. The average rainfall values (measured in meter) were rasterized by performing empirical Bayesian kriging interpolation, producing precipitation values for each pixel in the study area. The precipitation raster is presented in Fig. 14.

#### Distance from stream

**Fig. 15:**
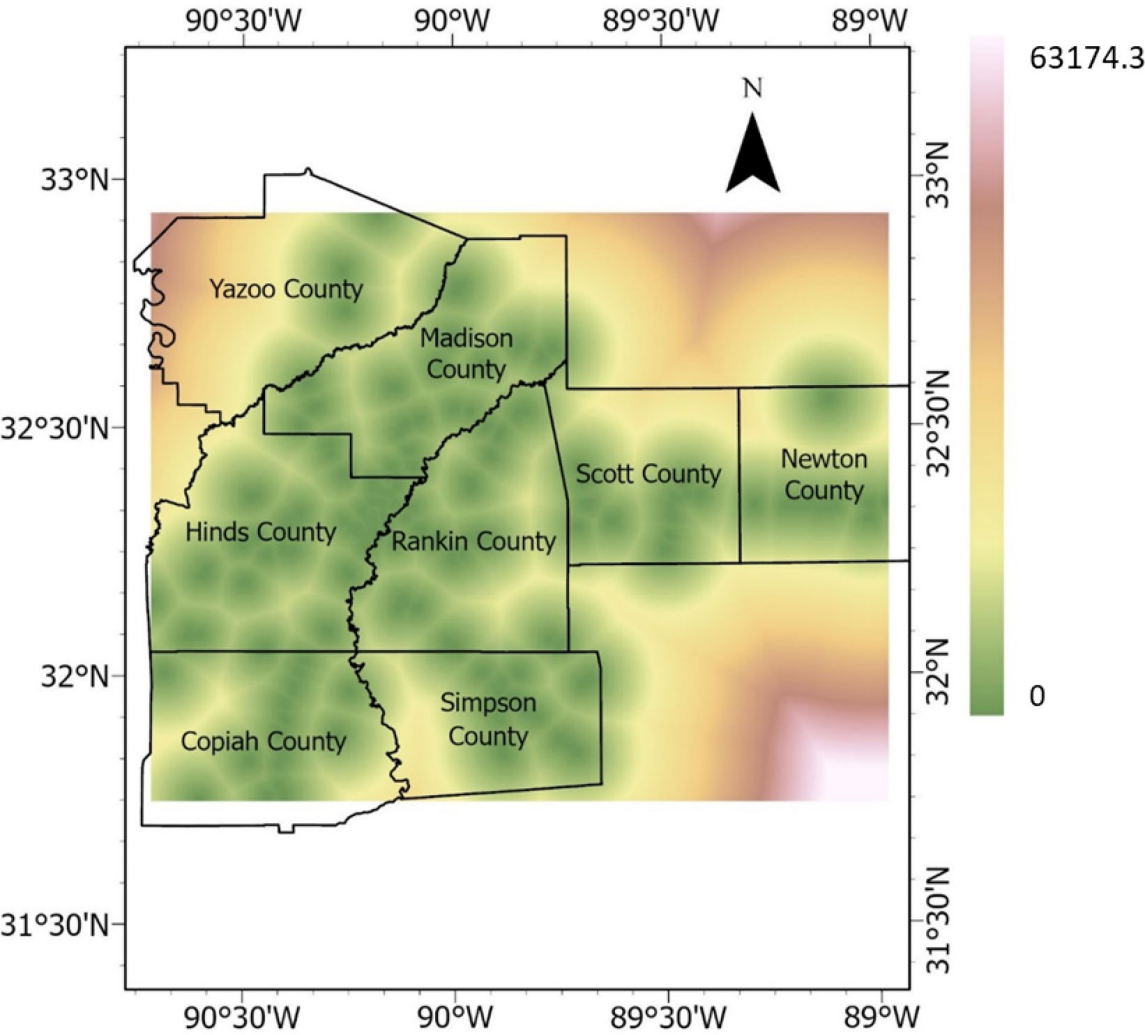
Distance from stream raster (meter). This spatial representation was derived from the DEM using flow accumulation and stream thresholding; the distance from the stream was then computed using the Euclidean distance. All these processes were performed by the first author (R.S.) using ArcGIS Pro software (Esri, version 3.0, www.esri.com/arcgis). The DEM used was from the aerial LiDAR data publicly available from MARIS (maris.mississippi.edu).

Distance from stream refers to the spatial measurement of a particular location or feature’s distance from a stream or river. Slopes and embankments near streams could be affected more due to soil saturation or changes in groundwater levels. Using the DEM, catchment areas were delineated in the study area to identify streams. Then, the distances based on the Euclidean method were calculated using ArcGIS, and the results were rasterized. The distance from the stream raster is presented in Fig. 15.

#### Water table depth and soil type

**Fig. 16:**
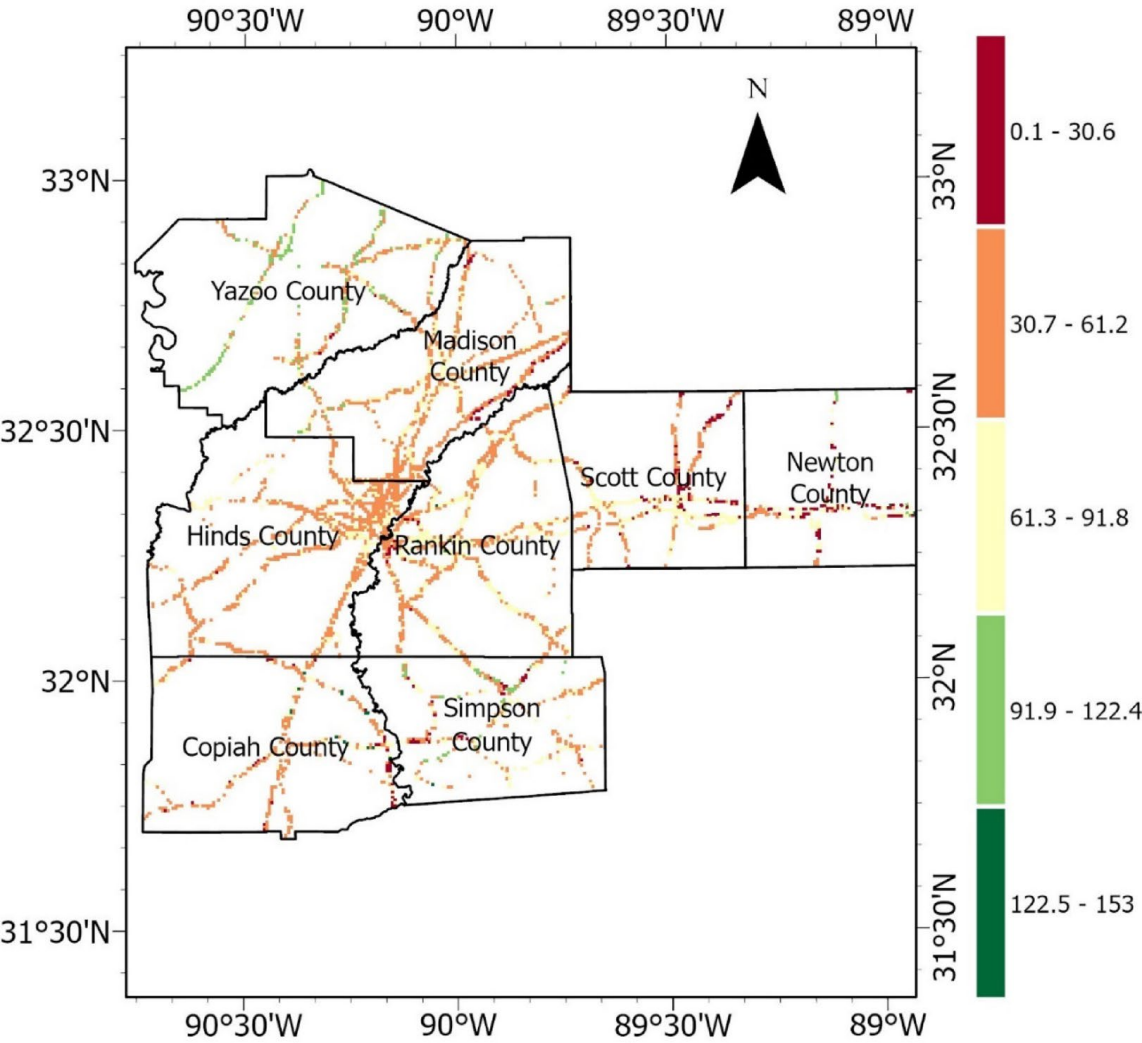
Groundwater table depth (centimeter) raster.

**Fig. 17:**
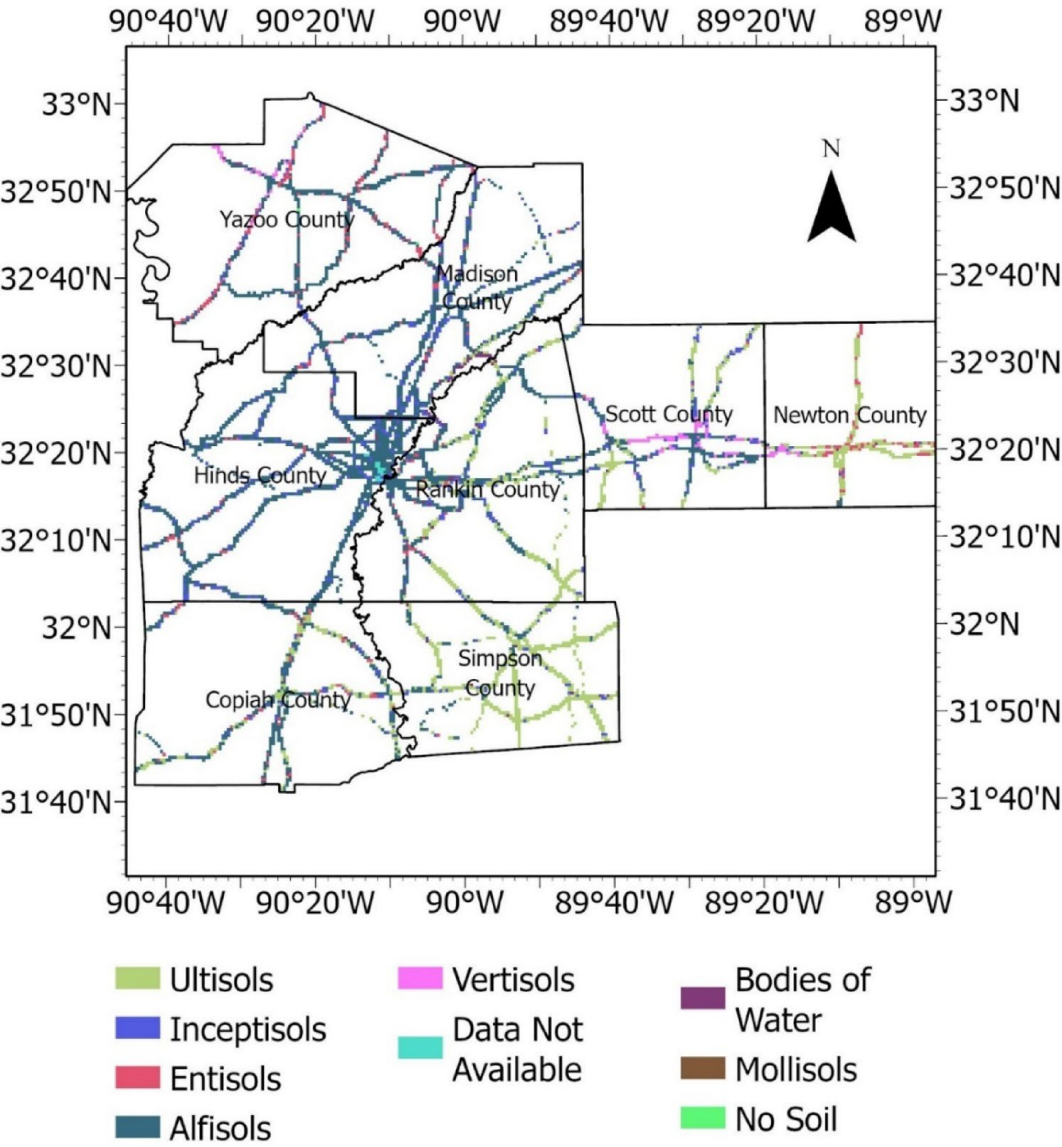
Soil type raster (ESRI Symbology nomenclature).

Groundwater is a crucial factor that impacts pore water pressure, effective shear strength, and soil density; therefore, the groundwater table depth was considered a causative factor of embankment failure. The USA Soils dataset was downloaded from the Living Atlas Library offered by ESRI. The groundwater table depth and soil type classified per ESRI symbology were extracted from the dataset, rasterized, and are presented in, Figs. 16, 17, respectively.

### Feature selection optimization

Feature selection plays a critical role in improving model performance for landslide and slope failure susceptibility mapping. Several feature optimization techniques were implemented in this study to identify the most relevant variables and eliminate noise or redundant information. Optimized feature selection helps prevent overfitting and enhances the model’s ability to better generalize across varied datasets^51^. In this study, three feature selection algorithms, Information Gain, Principal Component Analysis (PCA), and similarity matrix or correlation, were implemented to streamline the feature selection process. These algorithms have been successfully used to select optimal features for landslide prediction by researchers such as^51^ and^32^. Feature selection and dimensionality reduction methods vary in whether they use the target variable, their learning type, and their purpose.

#### Principal component analysis (PCA)

PCA is an unsupervised technique that does not rely on the target variable and is used to reduce dimensionality while retaining as much data variance as possible. PCA helps identify the components that explain the most variability in the dataset. PC1 almost always explains the most, since PCA orders components by decreasing explained variance. Features with high absolute loading values contributing to a principal component have more influence on the outcome. For example, in Table 2, PC1 explains 20% of the variance, and TWI and Precipitation have high absolute loadings and are the most influential causative factors.

**Table 2:** PCA results.

Principal component	Top contributing causative factors (PCA loading in parentheses)	Explained variance ratio
PC1	Aspect (|0.47|), TWI(|0.43|), Precipitation (|0.38|)	20%
PC2	Curvature (|0.6|), Plan Curvature(|0.51|), Profile Curvature(|0.55|)	17%
PC3 & PC4	Elevation (|0.64|), Distance from Streams (|0.61|), Soil Type Name(|0.41|)	24%
PC5 & PC6	NDVI, Soil Albedo, and Flow Accumulation	15%

#### Correlation

**Fig. 18:**
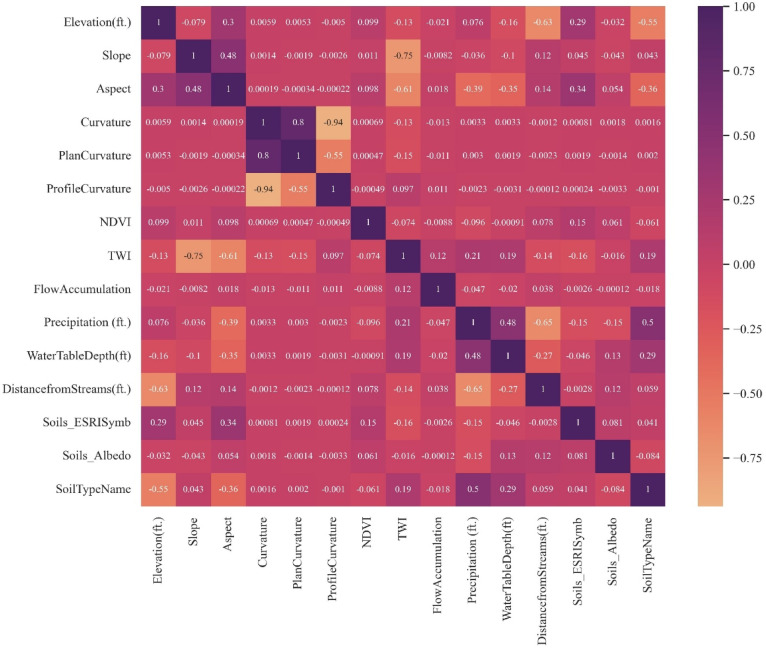
Feature correlation heatmap using pearson coefficient.

Correlation analysis using the Pearson coefficient, also known as a similarity matrix, is an unsupervised method that is a feature selection technique. It helps identify and remove redundant or highly correlated features. The Pearson correlation heatmap (Fig. 18) shows a strong correlation between curvature and plan curvature.

Several notable high correlations (positive and negative or inverse) were observed among the features. Elevation and distance from stream exhibited a relatively higher negative correlation of − 0.63, suggesting that higher elevations are generally farther from streams. Plan curvature and curvature showed a strong positive correlation of 0.80. In contrast, profile curvature and curvature were strongly negatively correlated at − 0.94, which makes sense because a positive profile curvature indicates upward curvature (concave-up). In contrast, negative profile curvature indicates downward curvature (concave-down). Additionally, topographic wetness index (TWI) showed strong negative correlations with slope (− 0.75) and aspect (− 0.61), which is expected given that TWI is derived using slope and catchment area, despite being used here as a hydrological index informing areas that can get wet.

In classification models (for example, RF, SVC, etc., used in this study) with categorical target variables, multicollinearity among features is generally less problematic than in linear regression models. While correlated features may dilute individual feature importance due to shared variance, this does not typically reduce model accuracy drastically. In such cases, the true predictive value may be distributed across correlated variables, making individual feature importance appear low, even though together they are highly influential to the model’s prediction. However, the importance of the feature can be understood through informed interpretation with the help of the correlation map of the feature similarity matrix presented in Fig. 18.

#### Information gain

**Fig. 19:**
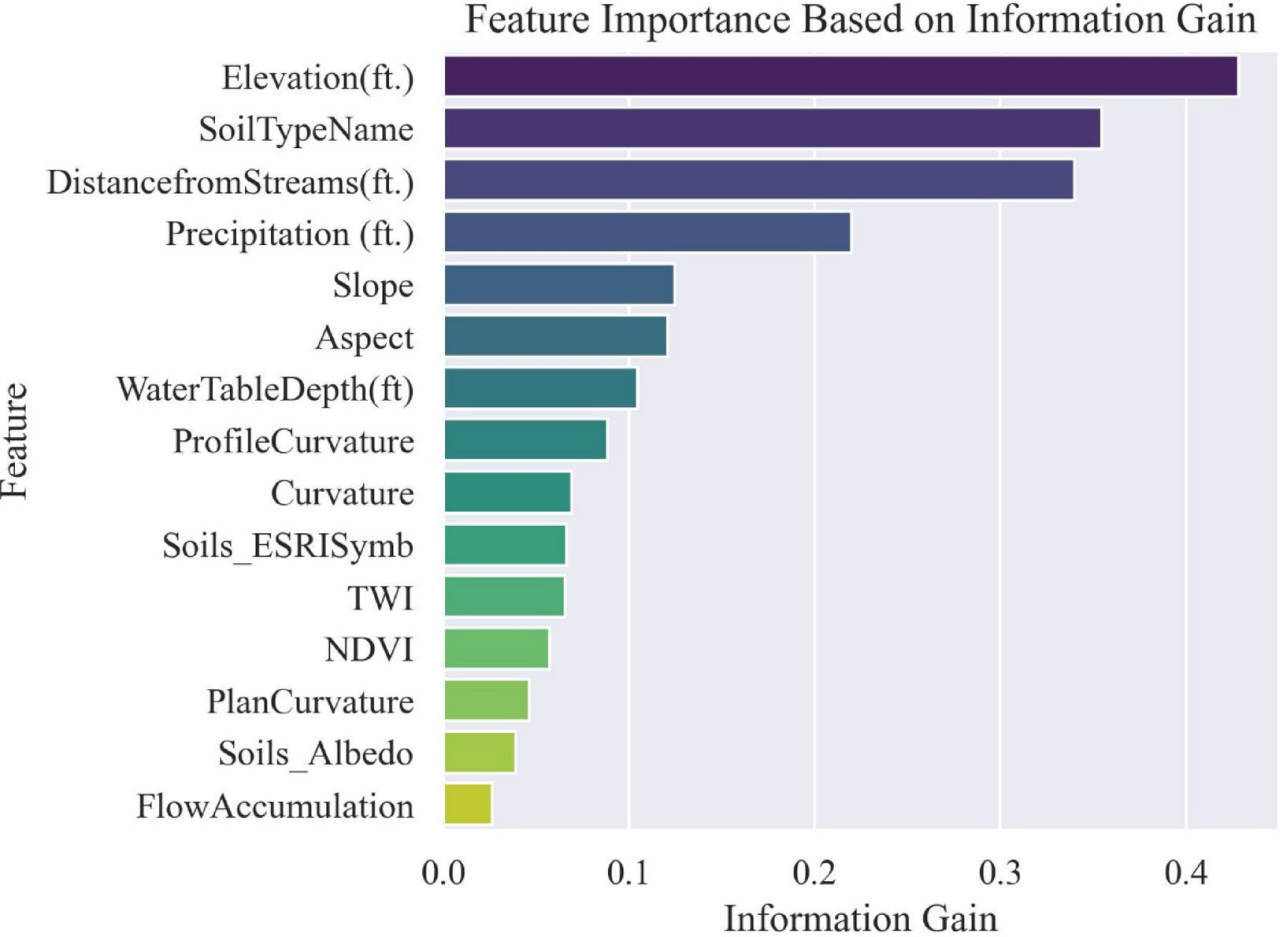
Information gain of the causative factors.

Information Gain is a supervised method that, unlike the prior two feature selection algorithms, includes the target to evaluate and select features that are most predictive of the outcome. It quantifies the extent to which a feature reduces uncertainty (entropy) about the target. The Information gain of all 15 features was evaluated with the target values, and the results in the chart format are presented in Fig. 19.

From PCA and information gain results, soil albedo and flow accumulation were found to have low absolute loadings (Table 2) and the lowest information gain (Fig. 19). Hence, they were dropped from the features dataset. Additionally, plan curvature was also removed from the feature list due to multicollinearity issues (Fig. 18) and low information gain (Fig. 19).

### Machine learning models evaluated

Several ML Models, namely, random forest, Naïve Bayes, support vector classifier, and logistic regression, were tested for their accuracy in classifying HWS failures.

#### Random forest (RF)

Random forest (RF) is an ensemble learning method. In this method, multiple decision trees are constructed in the training process that generate the output modes defined by the classification tasks. It is known for its robustness, high accuracy, and resistance to overfitting, making it suitable for complex datasets with numerous features. It combines the predictions of individual trees to produce a more reliable and stable classification, and the overall prediction is a combination of predictions from individual trees. The expression for the predicted class probability *P(yi)* for a sample *i* in a binary classification scenario is described in Eq. (3).

3 $$P\left({y}_{i}\right)=\frac{1}{N}{\sum }_{j=1}^{N}{P}_{j}({y}_{i})$$

where *N* is the number of trees in the forest, and *P*_*j*_*(y*_*i*_*)* is the predicted probability from the *j-*th tree for class *y*_*i.*_

Long et al.^32^ found that the RF model delivered high accuracy with satellite imagery and compared for automatic landslide detection over a ten-year period. RF was selected due to its proven reliable performance, and since it consistently performs better than other ML models for classification problems such as LSM^12,14,16,52^.

#### Naïve bayes (NB)

Naïve Bayes (NB) is a probabilistic classifier based on Bayes’ theorem with the "naïve" assumption of independence between features. It is computationally efficient and particularly effective for text classification and spam filtering. NB calculates the probability of each class of a given set of features and selects the class with the highest probability as the predicted label. The posterior probability, for a binary classification problem, $P({C}_{k}|X)$ for class ${C}_{k}$ given features X are computed using Eq. (4).

4 $$P\left({C}_{k}|X\right)= P\left(X\right)P\left(X|{C}_{k}\right)*P({C}_{k})$$

where $P\left({C}_{k}|X\right)$ is the likelihood, $P({C}_{k})$ is the prior probability of class ${C}_{k}$, and $P(X)$ is the evidence.

#### Support vector machine classifier (SVC)

Support vector machine classifier (SVC) is a supervised ML algorithm suitable for regression and classification problems. SVC works by identifying the hyperplane that best separates different classes in the feature space while maximizing the margin between them. SVC is effective in high-dimensional spaces and is capable of handling nonlinear relationships through the use of kernel functions. For a binary classification problem, a linear SVC finds the hyperplane represented by w*x + b = , where w is the weight vector, x is the input vector, and b is the bias. The decision function is given by Eq. (5).

5 $${\mathrm{f}}\left( {\mathrm{x}} \right) = {\mathrm{w}}*{\mathrm{x}} + {\mathrm{b}}$$

The predicted class is determined by the sign of f(x), with f(x) > 0 corresponding to one class and f(x) < corresponding to the other class.

#### Logistic regression (LR)

Logistic regression (LR) is a linear model estimating the probability of an instance belonging to a particular class and is suitable for binary classification problems. LR models the relationship between the dependent binary variable and one or more independent variables using the logistic function. It is simple, interpretable, and well-suited for scenarios with approximately linear decision boundaries. For binary LR, the logistic function (sigmoid) is used to model the relationship between the independent variables, X, and the log odds of the dependent variable, Y. The logistic function is defined as shown in Eq. (6).

6 $$P\left(Y=\frac{1}{X}\right)=\frac{1}{1+{e}^{-({\beta }_{0}+{\beta }_{1}{X}_{1}+..{\beta }_{n}{X}_{n})}}$$

where ${\beta }_{0},{\beta }_{1},..{\beta }_{n}$ are the coefficients.

#### Model performance metrics

The following metrics were used to test the model’s performance. Sklearn functions were used to calculate the metrics.

*Coefficient of Determination (R2_Score):* The Coefficient of Determination (R2_Score) is a key statistic used to measure how well a regression model predicts the dependent variable based on the independent variables. R2_score shows the proportion of variance in the target variable that the model can explain. The formula for the R2 score is given in Eq. (7). Essentially, it evaluates the fit of the model’s predictions to the actual data. The R2 value ranges from 0 to 1, where a score of 1 signifies a perfect prediction of the target variable, and a score of 0 means the model fails to account for any variance in the target.

7 $$R2\_Score = 1 - \sum\limits_{i = 1}^{n} {\frac{{(x_{i} - y_{i} )^{2} }}{{(x_{i} - x_{mean} )^{2} }}}$$

where y_i_ is the predicted value, x_i_ is the actual value, x_mean_ is the mean value of the true variables, and n is the total number of variables.

*Confusion Matrix:* A confusion matrix evaluates the performance of a classification algorithm in a set of data with known true values. It provides a detailed breakdown of the model’s predictions, highlighting the instances of correct and incorrect classifications. The confusion matrix helps assess the performance of a classification model and is particularly useful for binary and multiclass classification problems. It typically consists of four components: true positive (TP), when the model correctly predicts a failed area as failed; false positive (FP), when the model falsely predicts a non-failed area as failed; true negative (TN), when the model correctly predicts non-failed areas as non-failed; and false negative (FN), when the model falsely predicts failed areas as non-failed. Several more performance metrics that can be derived from the confusion matrix results are listed in Eqs. (8–12)

*Specificity:* Specificity is the True Negative Rate (TNR) and the formula is described in Eq. (8).

8 $$Specificity \, \left( {True \, Negative \, Rate \, \left( {TNR} \right)} \right):\frac{TN}{{TN + FP}}$$

9 $$False \, Positive \, Rate \, \left( {FPR} \right) \, = \, 1 - Specificity \, = \, 1 - TNR$$

*Precision: Precision measures the* accuracy of positive predictions by a model, indicating the proportion of true positives out of all predicted positives. In simple words, precision reflects the total number of correct positive predictions, which in this case are slope failure occurrences. It is described in Eq. (10).

10 $$Precision \, \left( {Positive \, Predictive \, Value} \right) \, = \frac{TP}{{TP + FP}}$$

*Recall:* Recall, also referred to as Sensitivity, is the True Positive Rate (TPR). It is a measure of the completeness of positive predictions. The ratio of correctly predicted positive instances to the total number of actual positive instances provides the recall. The formula for Recall or TPR is given in Eq. (11).

11 $$Recall \, \left( {Sensitivity \, or \, True \, Positive \, Rate \, \left( {TPR} \right)} \right) \, = \frac{TP}{{TP + FN}}$$

*F1_score:* The F1 score is the harmonic mean of precision and recall, offering a balanced evaluation metric when both false positives and false negatives are important. It is helpful when models can achieve high precision but low recall, or vice versa. The F1 score penalizes extreme imbalances between the two. A high F1 score indicates strong overall performance in identifying true positives without incurring excessive false positives. F1_score can be calculated using Eq. (12).

12 $$F1 \, Score \, = \frac{Precision*Recall}{{Precision + Recall}}$$

*Accuracy:* Accuracy measures the number of times a model’s predictions are correct, regardless of whether they are positive or negative predictions. In other words, it reflects the classifier model’s prediction correctness, encompassing true positive and true negative predictions. The method of calculating Accuracy is dividing total correct predictions by total predictions, as presented in Eq. (13).

13 $$Accuracy \, = \frac{TP + TN}{{TP + TN + FP + FN}}$$

*Receiver Operating Characteristic (ROC) Curve:* The ROC curve is a graphical representation that illustrates the performance of a binary classification model at various classification thresholds. The ROC curve assesses the trade-off between the true positive rate (sensitivity) and the false positive rate (1—specificity). The ROC curve is obtained by plotting two important parameters with false positive rates (1-specificity) on the x-axis and true positive rates (sensitivity value) on the y-axis.

*Area under Curve (AUC) Score:* The area under the ROC curve (AUC) serves as a key metric for evaluating the accuracy of a model. An AUC score of 1.0 indicates perfect accuracy, while a score closer to 0.5 suggests that the model performs only marginally better than random guessing, reflecting lower accuracy.

### Model development

**Fig. 20:**
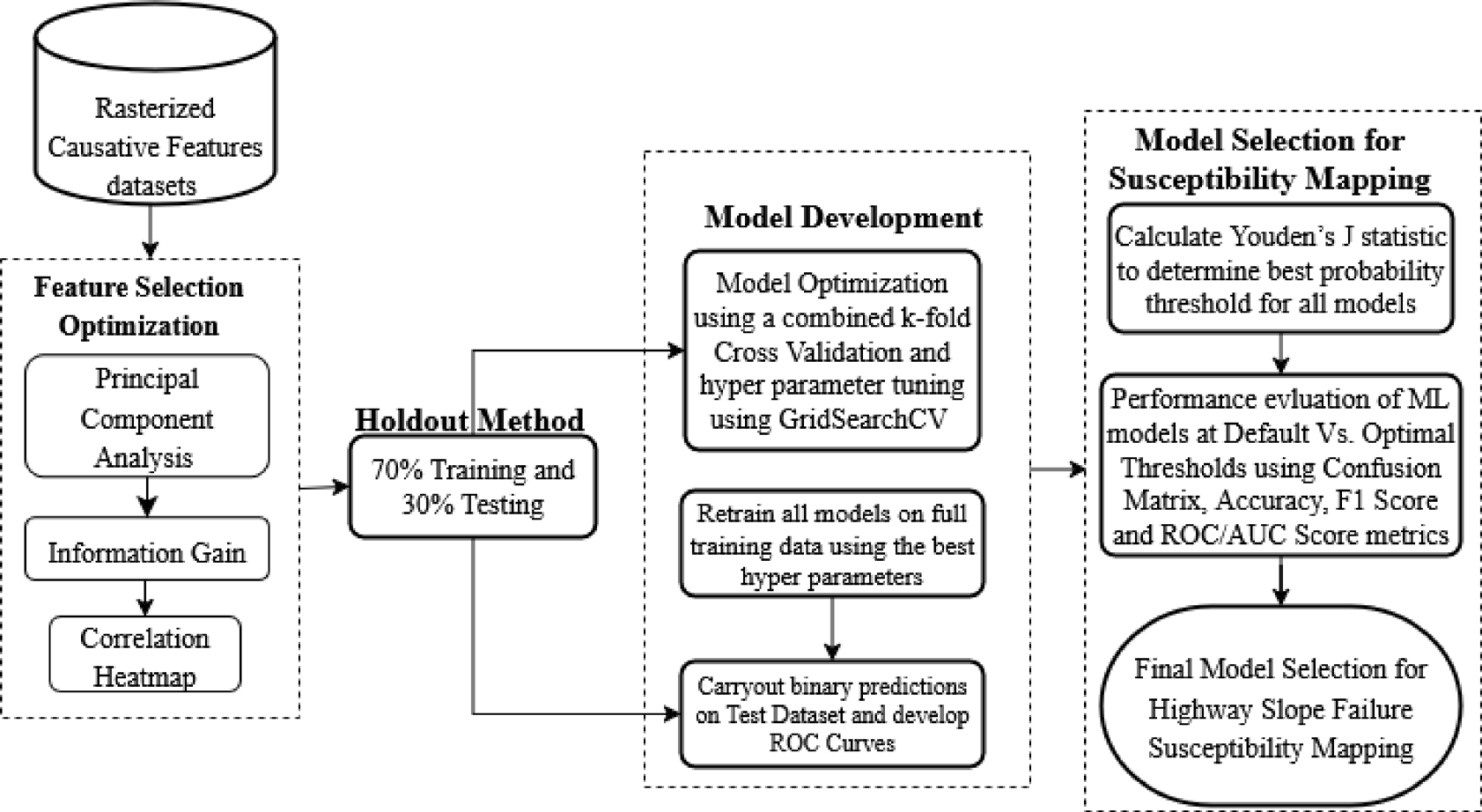
ML classification workflow for highway slope failure susceptibility mapping.

The model development, optimization, and selection were carried out as described in the workflow schematic presented in Fig. 20 for HWS Failure Susceptibility Mapping.

#### Holdout method

The holdout method was employed to develop the HWS failure susceptibility prediction models. The method involved splitting the dataset into two parts, 70% for training and 30% for testing. The dataset was randomly split into 70% for training the models and 30% for testing the model performance and checking for overfitting and accuracy. The train-test split method in the sklearn library was used to split the dataset randomly. The models were then trained on the training data to learn relationships between causative factors and target classification classes. Subsequently, the models were tested on the test set to evaluate their performance using metrics such as accuracy, precision, recall, and F1-score.

#### Model optimization

Hyperparameter tuning and k-fold cross-validation were performed jointly during the model training process to optimize predictive performance and enhance the generalizability of the models. A fivefold stratified cross-validation was implemented to ensure that the class proportions remained consistent across training and validation folds, thereby reducing the risk of biased model evaluation. To identify the best-performing configurations, each model architecture was subjected to Grid Search, a systematic and commonly used hyperparameter tuning approach^32^. This method evaluates all possible combinations of hyperparameter values within predefined ranges. By combining Grid Search with cross-validation, the evaluation of each hyperparameter setting was based on its average performance across all folds, thus ensuring a more robust and reliable selection process. Each ML model was evaluated using multiple hyperparameter combinations specific to the model. The full list of hyperparameter ranges explored for each model is summarized in Table 3.

**Table 3:** Summary of hyperparameter tuning within cross-validation folds.

Model	Hyperparameter	Description	Predefined Ranges Tested
RF	n_estimators	Number of trees	100, **200**, 500
	max_depth	Max depth of each tree	10, **20**, None
	max_features	Features to consider when splitting	"**sqrt**", “log2”, int, float
	class_weight	Weight for unbalanced classes	**None**, “balanced”
SVC	C	Regularization strength (trade-off margin vs error)	0.01, 0.1, 1, **10**
	kernel	Type of kernel function	linear’, '**rbf**', ‘poly’
	gamma	Kernel coefficient for ‘rbf’, ‘poly’, etc	**Scale**', ‘auto’, float
	class_weight	Handle imbalance	**None**, “balanced”
LR	C	Inverse of regularization strength	0.01, **0.1**, 1, 10
	penalty	Type of regularization	l2’, '**l1**'
	solver	Optimization algorithm	liblinear’, ‘lbfgs’, '**saga**'
	class_weight	Class imbalance handling	**None**, “balanced”
NB	var_smoothing	Smoothing parameter for Gaussian variance	1e-9, 1e-8, **1e-7**

NBmodels generally have few or no tunable hyperparameters. The **bold and underlined** values in the “Predefined Ranges Tested” Column represent the best hyperparameter values obtained from the tuned models, based on the results of fivefold cross-validation.

### Custom probability threshold for model optimization

It is equally important to minimize both false negatives and false positives when conducting a HWS failure susceptibility mapping. Overlooking susceptible areas could result in catastrophic failures, while misclassifying too many stable areas as high-risk could lead to inefficient use of already limited engineering budgets for geotechnical mitigations. Therefore, relying on a fixed threshold of 0.5, where a prediction is classified as slope failure if the probability is ≥ 0.5, and no failure otherwise, may not be ideal for accurately identifying HWS failure susceptibility.

To address this balance, Youden’s J was employed to determine the optimal threshold by jointly maximizing sensitivity and specificity, ensuring a balanced and effective classification methodology. Youden’s J is calculated per Eq. (14).

14 $${\mathrm{J}} = {\mathrm{Sensitivity}} + {\mathrm{Specificity}} - {1}$$

## Results

### Model performance results

#### Cross-validation results

The models implemented for failure susceptibility classification were evaluated by comparing their performance metrics. The unseen or 30% test data was used to obtain classification results and compare them with the ground truth data. Finally, the receiver operating characteristic (ROC) curves and the area under the curve (AUC) score were developed to evaluate the models’ performance.

Model performances during the cross-validation stage are presented in Table 4, which indicates the mean performance metrics: F1_Score, Accuracy, and AUC scores from k-fold cross-validation of models with the best hyperparameters. The results clearly indicate that RF was the best-performing model, with SVC coming in at a close second.

**Table 4:** Comparison of model performance from cross validation (CV) results.

Model	Best hyperparameter	Accuracy_CV	F1 Score_CV	AUC_CV
RF	n_estimators = 200; max_depth = 20; max_features = “sqrt”; class_weight = None;	0.9986	0.9986	1
SVC	C = 10; kernel = rbf; gamma = scale; class_weight = None;	0.9696	0.968	0.9928
LR	C = 0.1; penalty = l1; solver = saga; class_weight = None;	0.7973	0.7759	0.8724
NB	var_smoothing = 1E-7	0.7495	0.6813	0.8402

From the model optimization results, RF and SVC emerged as the top two best-performing models, followed by LR nd NB respectively. It is important to note that the LR and NB performance metrics improved significantly after feature selection optimization.

Subsequently, the full dataset was freshly split into 70% for training and 30% for testing, and new models (RF, SVC, LR, and NB) were built with the best hyperparameters and trained on the full training dataset.

The performance of the trained models was assessed using their results on unseen test data, with metrics such as F1 score, accuracy, and AUC utilized for evaluation.

To enhance model performance further, classification outcomes using the default probability threshold of 0.5 were evaluated against results obtained with optimal custom thresholds.

#### Custom probability thresholds

Using Youden’s J method, the optimum custom threshold values for each model is presented in Table 5

**Table 5:** Optimum Probability Threshold using Youden’s J Statistic.

Model	Default threshold	Optimum threshold
RF	0.5	0.7576
NB	0.5	0.3987
SVC	0.5	0.2936
LR	0.5	0.3331

Thresholds that maximized Youden’s J were selected as optimal thresholds for classification.

**Fig. 21:**
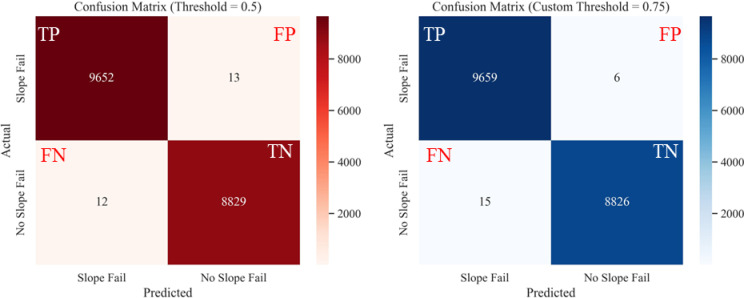
Confusion matrices from RF classification on test dataset for: (**a**) default threshold (0.5), (**b**) optimal custom threshold (0.75).

**Fig. 22:**
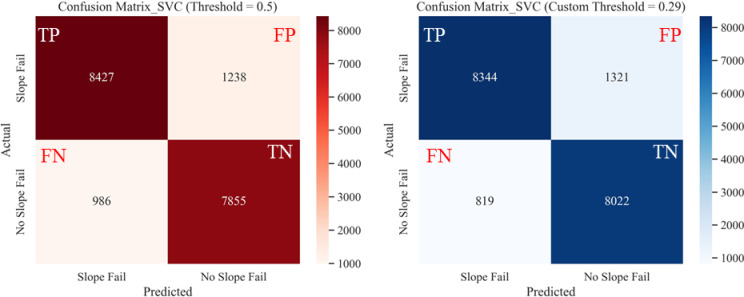
Confusion matrices from SVC classification on test dataset for: (**a**) default threshold (0.5), (**b**) optimal custom threshold (0.29).

**Fig. 23:**
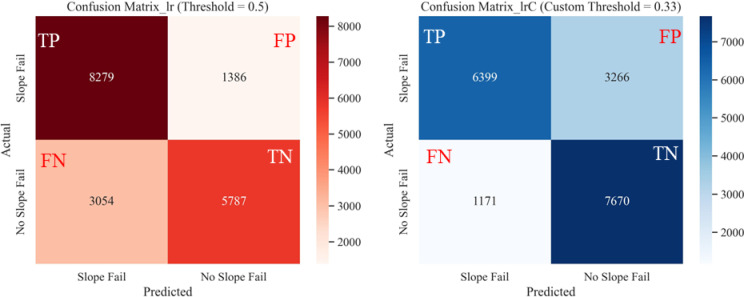
Confusion matrices from LR classification on test dataset for: (**a**) default threshold (0.5), (**b**) optimal custom threshold (0.33).

**Fig. 24:**
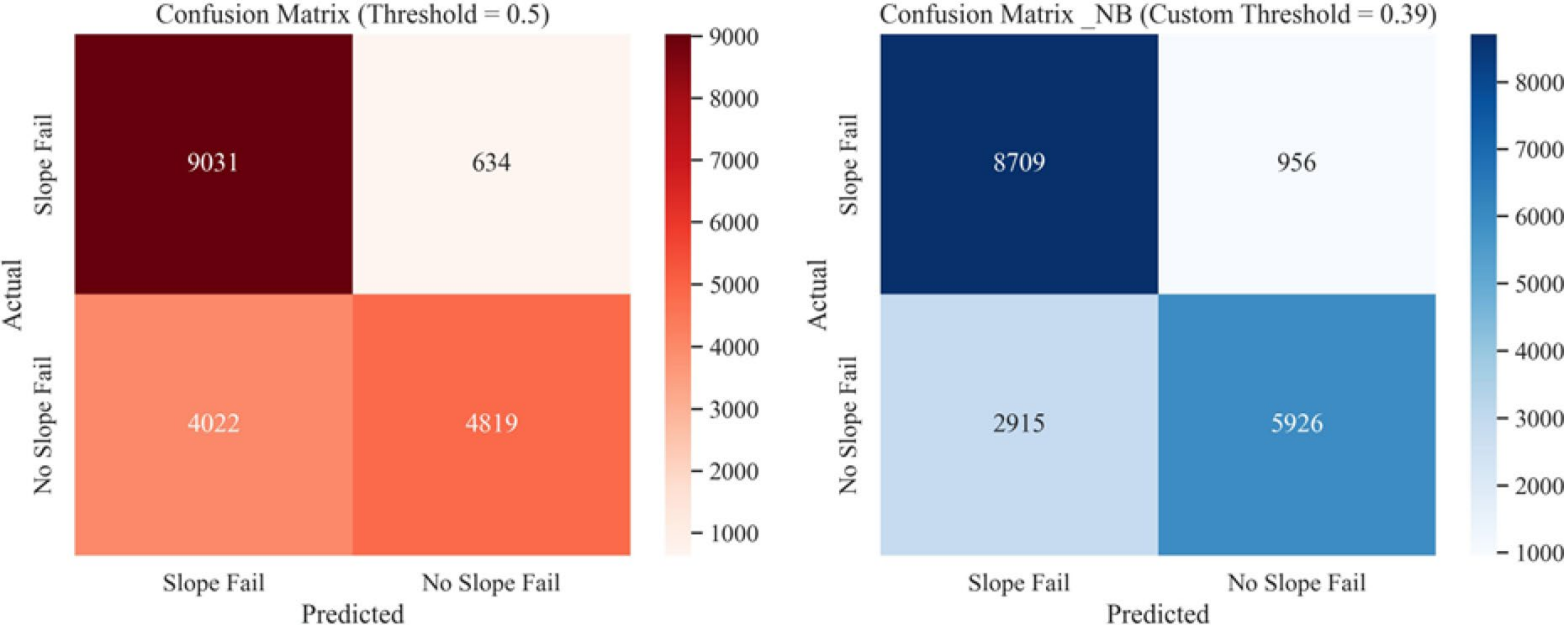
Confusion matrices from NB classification on test dataset for: (**a**) default threshold (0.5), (**b**) optimal custom threshold (0.39).

Confusion matrices were developed from the results obtained from the classifier models and were further evaluated for default threshold versus optimal threshold values. The confusion matrices of ML classification models, RF, SVC, LR, and NB, are presented respectively in Figs. 21, 22, 23, 24.

**Fig. 25:**
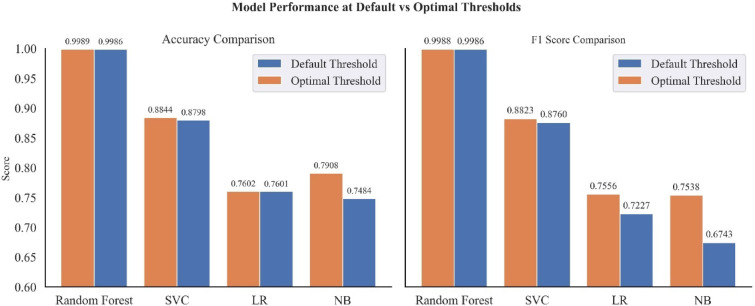
Performance metrics of ML models at Default Vs. Optimal Thresholds: (**a**) accuracy score comparison, (**b**) F1 score comparison.

Comparisons of performance metrics of the different models at default and optimal probability thresholds are presented in Fig. 25. The accuracy and F1 scores of the four models (RF, SVC, LR, and NB) were compared using both the default threshold (0.5) and the optimal threshold (0.75 from Youden’s J method). The optimal probability threshold improved the performance metrics of each model. Although there was not much room for improvement on RF and SVC models, which performed well even with the default probability threshold of 0.5, the custom threshold still produced better classification results. On the other hand, LR and NB model performance improvements were much pronounced when using custom thresholds versus the default thresholds.

#### Receiver operating characteristic (ROC) curve

**Fig. 26:**
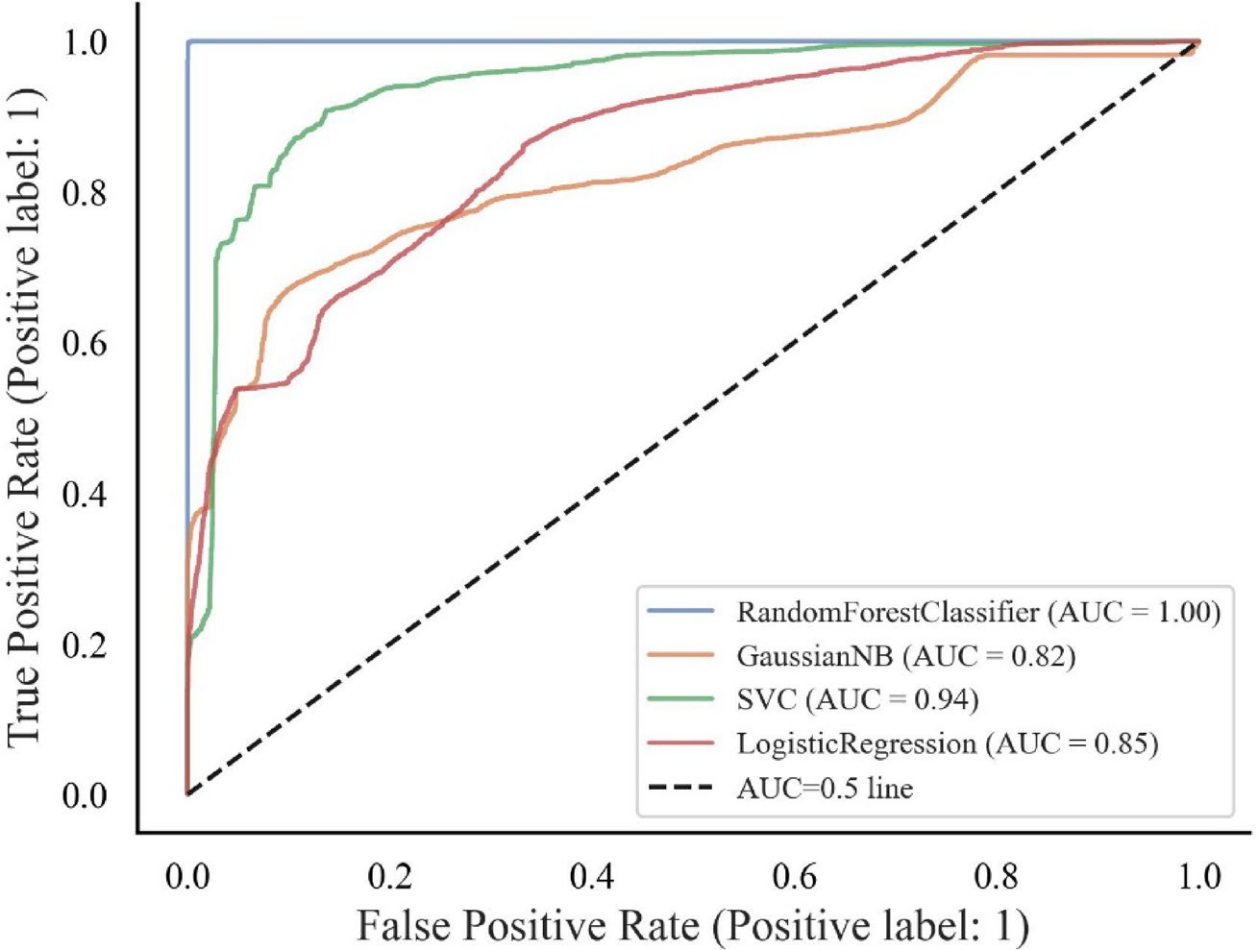
ROC curve and AUC for the ML models.

The ROC curve is a graphical representation that illustrates the performance of a binary classification model at various classification thresholds. The ROC curve assesses the trade-off between the true positive rate (sensitivity) and the false positive rate (1-specificity). The ROC curve is obtained by plotting two important parameters with false positive rates (1-specificity) on the x-axis and true positive rates (sensitivity value) on the y-axis. A model that performs no better than random chance would have an ROC curve along the diagonal line (from the bottom-left corner to the top-right corner). A model with a higher area under the curve (AUC), closer to 1, generally indicates better classification for the set threshold. A comparison of ROC curves for all ML models tested in this study is presented in Fig. 26 which clearly indicates that RF is the best-performing model with the highest AUC (1.0). SVC was the next best performing model with an AUC of 0.94. Based on the evaluation of the four ML models, RF performed the best and hence was chosen to predict HWS failure susceptibility.

### HWS failure susceptibility mapping

#### Random forest classification results

Due to the high resolution of the raster data (0.91 m × 0.91 m), the entire 8-county study area generated an immense amount of data (450 million rows or pixels. Processing to classify each pixel became extremely expensive, computationally. As a result, the HWS failure susceptibility prediction was conducted on a focused area of interest (AOI) of the national highway corridor along I-55 and I-20 in the Jackson, MS metro area. A major portion of this AOI was previously not used during model development, so the trained RF model was agnostic.

**Fig. 27:**
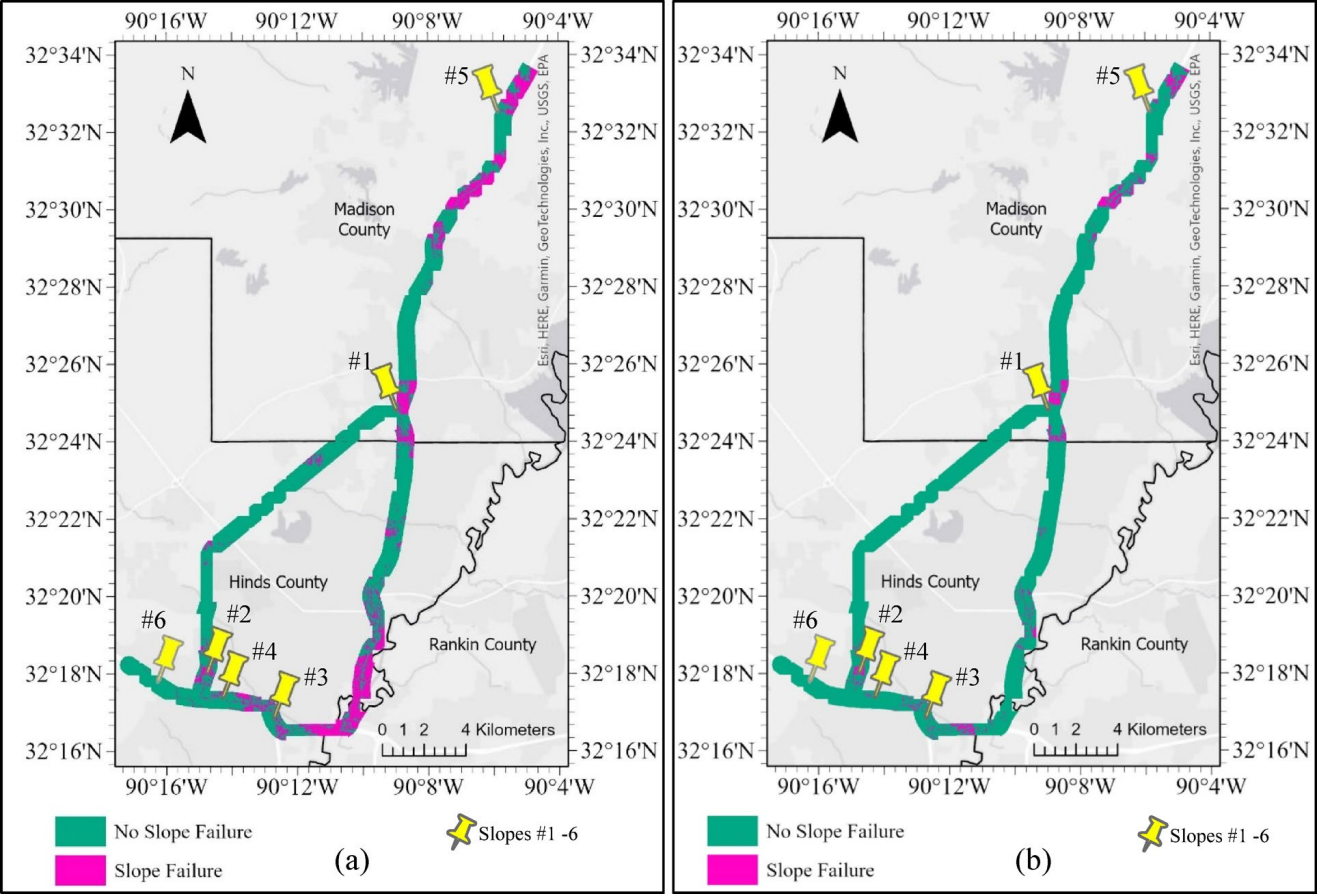
Highway slope failure susceptibility maps with binary classification for AOI in central MS produced by RF Model using: (**a**) default threshold of 0.5, and (**b**) custom optimal threshold of 0.75.

Additionally, HWS failure susceptibility maps (HWS-FSM) were generated using the trained classification model, RF, under two conditions. The two conditions were thresholding strategies: first, using the default threshold (0.5), and second, using an optimal custom threshold (0.75) derived from Youden’s J statistic to maximize classification performance. These probabilities were then classified into binary susceptibility maps using both the default and custom optimal thresholds as presented in Fig. 27a,b, respectively. These susceptibility maps with binary classifications indicate highway failure susceptibility. The default threshold provided a baseline binary classification, while the custom threshold offered a more balanced trade-off between false positives and false negatives. For instance, the HWS_FSM based on the default threshold (Fig. 27a) clearly indicates higher areas with failure classification. However, the optimal custom threshold HWS-FSM (Fig. 27b) has fewer areas classified as potential failures.

Six HWS previously studied by the authors with known conditions were used to validate the RF susceptibility map. These six highway slopes were excluded from model training and testing. Both the default threshold and custom threshold HWS failure susceptibility maps indicated that the slopes 2,3, and 4, which had previously experienced failures and exhibited signs of impending movement, corresponded precisely with areas identified as susceptible to failure on the map. Slope 1 was found to be less susceptible to failure. Conversely, Slope 6, which has remained intact and historically aligned with regions deemed not susceptible to failure.

**Fig. 28:**
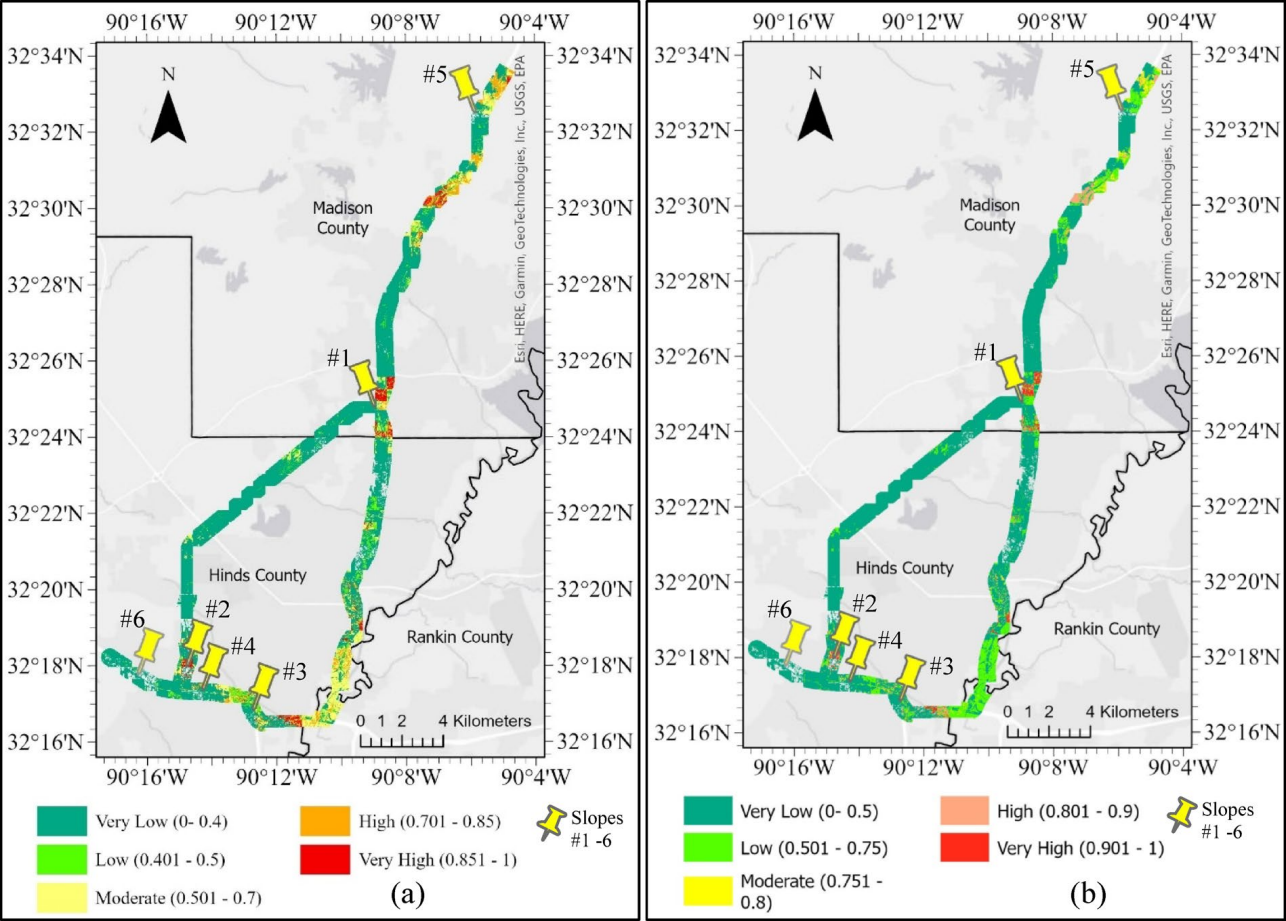
HWS failure susceptibility maps with five categories for AOI in central MS counties, produced by the trained RF Model using: (**a**) default threshold of 0.5, and (**b**) custom optimal threshold of 0.75.

Furthermore, two more susceptibility maps were developed by classifying the predicted probabilities into five categories – very low, low, moderate, high, and very high, using the default threshold (0.5) and optimal custom threshold of 0.75 derived from Youden’s J statistic. Instead of binary classification, this approach retained the full range of model-predicted probabilities, allowing for more granular mapping of landslide risk. The refined version of the HWS failure susceptibility maps with the five classification categories prepared applying the default and custom optimal thresholds are presented in Fig. 28a,b, respectively. The classification bins were initially defined based on natural breaks in the probability distribution, but then were slightly adjusted manually for better delineation and consistent interpretations across maps. This method allowed susceptibility to be expressed on a continuous risk spectrum, facilitating better risk communication and prioritization of interventions based on severity.

Comparing the outputs of the default and custom threshold classification revealed that adjusting the classification threshold significantly improves the model’s ability to distinguish susceptible and non-susceptible areas. The default-threshold-based classification provides a conventional midpoint split at a probability of 0.50. However, the custom-threshold-based classification improves the model sensitivity(or recall) and specificity by shifting the cutoff to the optimal decision point identified from the ROC curve for the RF model. This approach enhances the reliability of infrastructure asset management by reducing misclassification and aligning the model’s predictions with real-world slope failure risks.

#### Sensitivity analysis and feature importance

**Fig. 29:**
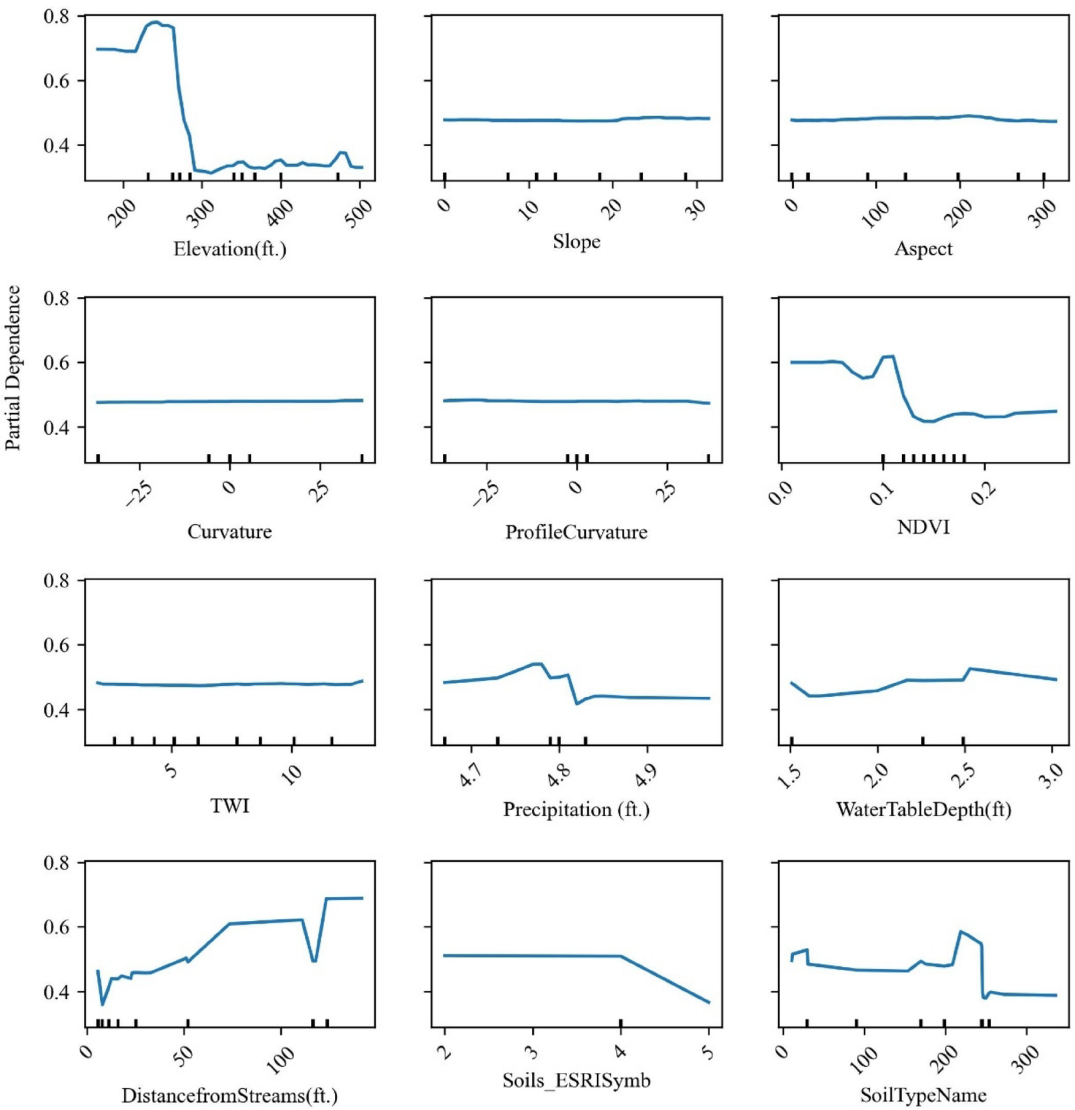
Partial Dependence Plots (PDPs) showing the effects of individual features on the predicted probability of HWS failure occurrence based on the trained RF classifier. The Y-axis represents the average model response (partial dependence), while the X-axis shows the feature values.

To further evaluate the influence of different features on the prediction, a sensitivity analysis was conducted on the leading RF Model. Partial Dependence Plots (PDPs) were generated to illustrate how predictions shift as individual features are varied, while all other variables remain constant. PDPs offer valuable insight into how ML models respond to changes in specific inputs, making them especially helpful for interpreting sensitivity in LSM. The goal of this analysis was to assess how responsive the model’s predictions are to each input variable. Figure 29 presents the PDPs for the top-performing RF model. The plots illustrate the influence of each feature on HWS failure susceptibility while holding all other variables constant.

The partial dependence analysis revealed that elevation, NDVI, distance from streams, and soil type are key predictors influencing landslide susceptibility. The model shows a sharp decline in predicted probability above ~ 250 ft elevation, indicating higher risk at lower elevations. This corroborates the field evidence where the toes of the slope experience more failures than the slope crest. Similarly, areas with low NDVI values (i.e., sparse vegetation) are associated with elevated risk, while increasing distance from streams significantly reduces susceptibility, likely due to reduced toe erosion and saturation. Certain soil types also show clear variation in response, suggesting a notable influence on slope stability. In contrast, features such as slope, curvature, aspect, and precipitation exhibited relatively flat PDPs, implying they are not key drivers of failure or could be correlated with other influential features within the dataset, which is completely plausible.

**Fig. 30:**
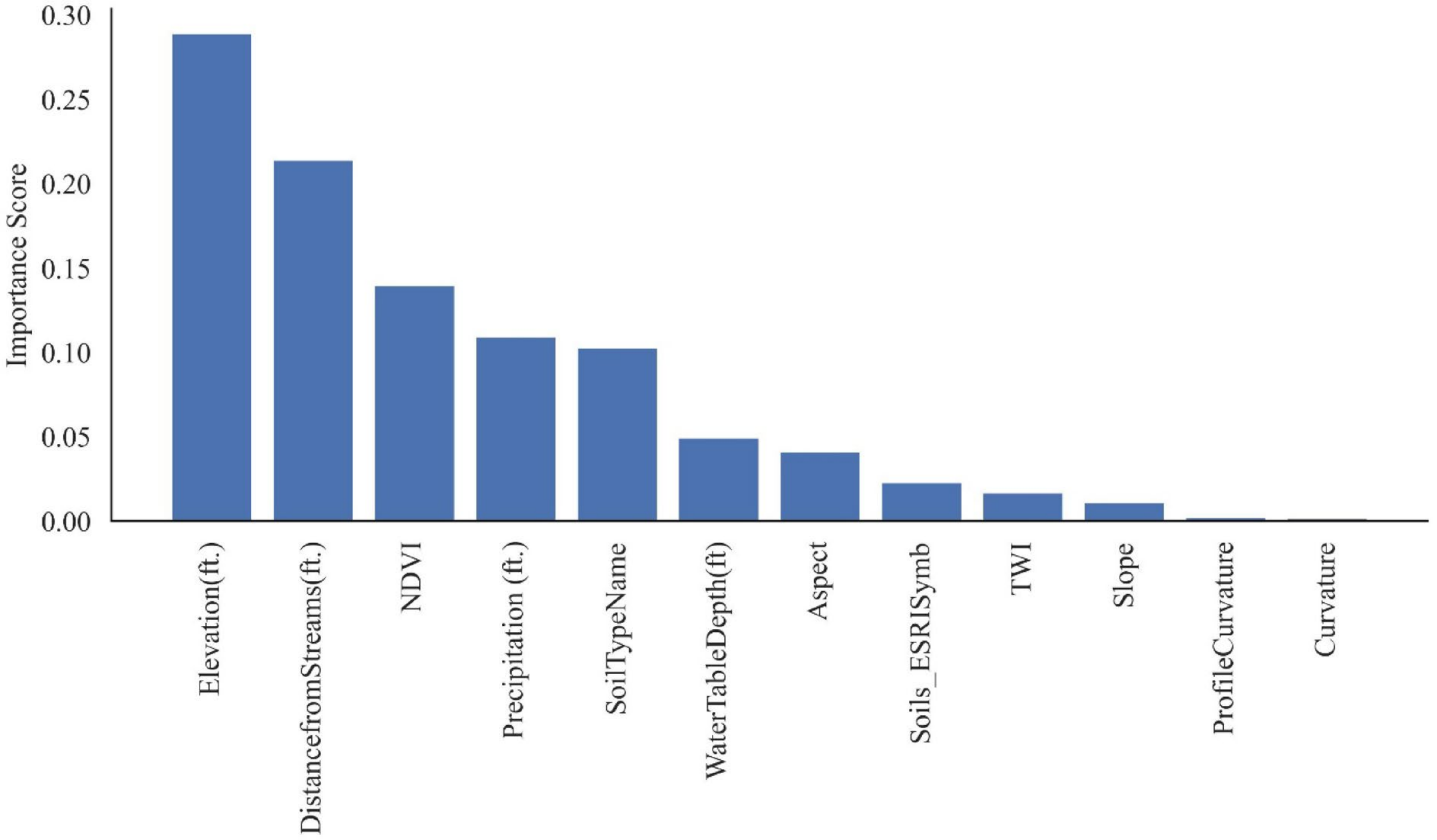
Random forest feature importances.

Furthermore, feature importance metrics were derived from the RF model’s classification results and presented in Fig. 30. The findings from the PDPs are further corroborated by the feature importance metrics presented in Fig. 30. The top five influential causative factors identified were elevation, distance from streams, NDVI, precipitation, and soil type. The presence of expansive clay soils across the study area, which are highly susceptible to volumetric changes during wet and dry weather cycles, likely contributes to the importance of soil type as a predictive feature.

## Discussion

The RF model emerged as the most effective classifier in our study, boasting an impressive area under the curve (AUC) of 1.0. This metric, indicative of the model’s ability to distinguish between positive and negative instances, underscored the robust performance, superior precision, strong recall, and f-1 score metrics of the RF algorithm, solidifying its status as the top-performing model in our analysis. Upon delving into the specifics of predicting HWS failures, the RF model pinpointed the four most influential factors: elevation (in the first place), closely followed by distance from a stream, the NDVI, and precipitation. These findings shed light on the critical variables contributing to the vulnerability of HWS and provide actionable insights for effective risk mitigation strategies.

In this study, rigorous model optimization was performed, including feature selection optimization, optimum custom threshold selection, hyperparameter tuning, and k-fold cross-validation. This integrated approach of optimal feature selection and hyperparameter tuning helped boost predictive performance and generalizability and provided insights into key factors influencing landslides^51^. It is important to use the optimal group of features to enhance model classification performance^32^. Feature selection helped identify the most relevant variables, reducing noise and overfitting, while hyperparameter tuning controlled model complexity and improved generalization to unseen datasets.

Furthermore, a custom optimal threshold was deemed necessary since the goal was to have a good balance between false negatives (missing a hazard-prone slope) and false positives (over-predicting slope failures to limit spending on costly resources). The selection of a threshold value of 0.75 for classifying HWS failure susceptibility reflects both the nuances of the dataset and the characteristics of the RF model. For instance, if HWS failures are a rare occurrence in the dataset, the model tends to assign low probabilities to most samples, necessitating a higher threshold to maintain specificity and avoid false positives. As a result, a higher threshold was found to be optimal, with the model requiring a strong signal before labeling a case as positive.

The sensitivity study, including PDP and feature importance analyses, identified elevation, distance from streams, NDVI, precipitation, and soil type as the top predictors of HWS failure. The significance of soil type aligns with the prevalence of expansive clay soils in the study area, which are prone to shrink-swell behavior under varying moisture conditions. While precipitation ranked highly in feature importance, its limited influence in PDPs suggests that its effect may be more pronounced when interacting with other variables rather than acting independently.

Building on these insights, the established failure susceptibility modeling methodology proved effective in evaluating unstable slopes and embankments to support geotechnical asset management. This methodology, validated by the high performance of the RF model, serves as a valuable tool for identifying potential failure-prone areas and guiding targeted interventions to enhance infrastructure resilience.

### Integration of HWS failure susceptibility mapping into GAM framework

The MAP-21 Act (2012) required all US state transportation agencies to adopt *risk-based asset management plans* for the National Highway System^53^. In response, most agencies implemented Transportation Asset Management (TAM) programs focused primarily on pavements and bridges. However, Geotechnical Asset Management (GAM) remains underdeveloped, with HWS often not included in an asset management program^9,54–56^ due to multiple reasons, possibly including limited funding, resources, and awareness.

Despite the proven potential of ML for failure prediction, such techniques are rarely applied to HWS. This study addresses that gap by introducing a scalable, data-driven approach for HWS failure susceptibility mapping using ML. The proposed method enables rapid screening of large geographic areas with minimal resources, making it particularly useful for state agencies operating under budget constraints. By identifying the at-risk critical infrastructure assets such as HWS, this approach can serve as a critical first step in prioritizing geotechnical assets for more detailed assessment.

When high-susceptibility hotspots are identified, agencies can deploy targeted sensing technologies such as LiDAR and UAV-based photogrammetry to evaluate surface deformation. If these assessments indicate ongoing or potential instability, they can be followed by geophysical and geotechnical investigations to evaluate subsurface conditions and precisely locate failure mechanisms. This methodical, tiered investigation process enables agencies to allocate resources more efficiently and take proactive mitigation measures, when necessary, especially for assets critical to intermodal transportation networks. This tiered approach supports timely, data-driven decisions and cost-effective risk mitigation.

**Fig. 31:**
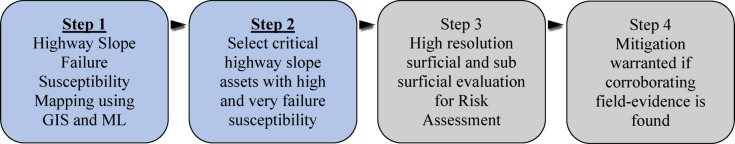
Integrate HWS susceptibility into GAM and infrastructure risk assessment framework.

Additionally, the trained model can be embedded in a smart GAM framework, offering continuous performance monitoring and early warnings. Figure 31 outlines how susceptibility mapping can be integrated into GAM and infrastructure risk management workflows.

### Limitations

Despite a comprehensive evaluation of the study area, the study still has several limitations. For instance, the model’s accuracy is dependent on the quality and resolution of input datasets, including the DEM used to generate several causative factors and NDVI generated from coarse Landsat satellite data, and rainfall from daily average values, which may vary spatially. The landslide inventory used for training may be incomplete or biased toward recent or accessible events, potentially affecting generalizability. These static causative factors do not capture the temporal variability of triggering factors like intense rainfall within short durations or land cover changes. Binary outputs may oversimplify susceptibility in complex terrain conditions, though threshold optimization improves classification.

Additionally, despite the use of PDPs and variable importance analysis, feature interactions may not be fully captured, and model interpretability remains limited. Critical infrastructure-specific details such as drainage and slope reinforcement are not included, which may influence slope stability. Lastly, the model may be region-specific and may not transfer well to other geographic contexts without recalibration, but this needs to be further evaluated.

### Future work

This study highlights the growing application of ML in long-term geomorphological monitoring and disaster risk assessment, demonstrating how models like RF can extract insights from satellite data and provide scalable methods for analyzing landslide evolution from various natural hazards. More avenues will be explored to implement this methodology tailored for other atmospheric forcings, including seismic events, hurricanes, extreme rain, and so on. Furthermore, the trained model’s robustness can be further assessed by applying it to HWS in different geographic regions, enabling evaluation of its transferability and generalizability across different terrains.

Future research will focus on developing hybrid ML models that integrate optimization techniques widely used in landslide susceptibility mapping (LSM), such as nature-inspired algorithms described by Liu et al.^57^. These approaches will be used to enhance model performance and predict displacement patterns more accurately.

Additionally, another area of exploration will include comparative analysis between slope unit and raster unit mapping approaches^23^, as well as expansion of the dataset to support robust sensitivity analyses. These analyses will assess the impact of individual and combined features on model predictions. Future models will incorporate input variables derived from InSAR and LiDAR observations, such as ground motion, time series deformation, and settlement, along with lithological, hydrological, and environmental data. The goal is to develop high-resolution susceptibility maps that are directly applicable to build environments and transportation infrastructure, enhancing early warning systems and infrastructure resilience.

## Conclusion

Geotechnical assets such as highway embankments and slopes (HWS) play a critical role in the stability and functionality of transportation infrastructure, but are often underrepresented in Transportation Asset Management (TAM) programs. The early identification and mapping of vulnerable HWS are vital for infrastructure resilience. This study developed a geographical information system (GIS)-based HWS failure susceptibility mapping framework by adapting established landslide susceptibility mapping techniques commonly applied to natural or cut hillslopes. Supervised ML models, including Random Forest (RF), Support Vector Classifier (SVC), Logistic Regression (LR), and Naïve Bayes (NB), were trained using remote sensing-derived DEMs and rasterized geotechnical, geomorphological, and hydrological features.

Among the models tested, RF demonstrated superior performance across all evaluation metrics, with AUC, F1 score, and Accuracy scores of 1.0 each. SVC came in second with AUC score = 0.94, F1 and Accuracy scores = 0.88 each, for both default and custom probability thresholds. Feature reduction through PCA and Information Gain further enhanced model efficiency by removing low-impact causative factors (features) such as soil albedo, flow accumulation, and plan curvature. Comparisons using default probability threshold (0.5) and a custom optimal threshold (0.75), derived via Youden’s J statistic, showed improved classification accuracy across all models, particularly for SVC, LR, and NB, confirming the value of threshold tuning in susceptibility mapping. The resulting susceptibility maps allow for the prioritization of high-risk assets across large areas with limited resources, improving the allocation of mitigation efforts. The top five influential causative factors identified were elevation, distance from streams, normalized difference vegetation index (NDVI), precipitation, and soil type. The presence of expansive clay soils across the study area, which are highly susceptible to volumetric changes during wet and dry weather cycles, likely contributes to the importance of soil type as a predictive feature. Although the RF model identified precipitation as an important feature for classification, the Partial Dependence Plots (PDPs) suggested a weaker individual influence. This result is somewhat counterintuitive, as precipitation is widely recognized as a key driver of landslides and ground movement. This discrepancy may indicate that certain factors exert greater influence through interactions or combinations, rather than as standalone variables.

Additionally, validation using six known HWS sites demonstrated strong spatial agreement between model predictions and observed failure conditions. This study introduced an innovative data-driven approach that integrates ML, remote sensing, and geospatial analysis to modernize traditional asset monitoring. Unlike conventional methods that rely heavily on historical failures or manual inspections, the proposed framework enables automated, scalable, and proactive geotechnical asset management (GAM). By incorporating both probabilistic classification and optimized thresholds, the proposed methodology enables early-warning prioritization, targeted inspections, and more nuanced decision-making. These benefits can significantly enhance infrastructure maintenance and planning. Moreover, HWS failure susceptibility modeling will streamline geotechnical asset risk assessment and support long-term strategies for managing transportation infrastructure.

## Data Availability

The datasets used and/or analyzed during the current study will be made available from the corresponding author on reasonable request.
